# Quantitative Multiplex Digital PCR with Fluorescence-Encoded Nanoreactor Beads

**DOI:** 10.1021/acs.analchem.6c00259

**Published:** 2026-05-18

**Authors:** Stephan Hubold, Lea Kanitz, Oliver Lemuth, Ines Engelmann, Susanne Toepfer, Susanne Brandes, Theresa Liebe, Friederike Fritsch, Sascha Braun, Martin Reinicke, Ralf Ehricht, Eugen Ermantraut

**Affiliations:** † BLINK AG, Bruesseler Strasse 20, 07747 Jena, Germany; ‡ Leibniz Institute of Photonic Technology, Member of the Research Alliance “Leibniz Health Technologies” and the Leibniz Centre for Photonics in Infection Research (LPI), Albert-Einstein-Straße 9, 07745 Jena, Germany

## Abstract

Syndromic PCR panels are becoming a pivotal diagnostic tool in precision medicine. Yet, the design and validation of such test panels is cumbersome, and the integration of new markers typically necessitates complete panel revalidation, a challenge that becomes even more complex for quantitative assays. Here, we introduce a novel multiplex digital PCR strategy utilizing fluorescence-encoded magnetic Nanoreactor Beads (femNRB), enabling cross-reactivity-free detection and absolute quantification of multiple genetic markers within a single sample. These uniquely encoded nanoreactor beads are reversibly conjugated to target-specific primers and probes, allowing the formation of highly adaptable panels composed of multiple PCR assays. As proof of concept, we assembled and validated a 10-assay panel targeting species-specific markers for common nosocomial pathogens. We subsequently expanded this panel with six additional markers for antibiotic resistance and virulence, systematically evaluating both configurations. Remarkably, without further optimization, both panels demonstrated equivalent analytical performance, underscoring the robustness and scalability of this method. This approach operates without complex microfluidic hardware and represents a diagnostic multiplex digital PCR platform that enables seamless assay integration while maintaining uncompromised quantification across variable sample concentrations. By eliminating the need for recalibration, this method substantially simplifies assay expansion, providing a powerful, modular solution for developing and upgrading diagnostic test panels with minimal effort.

## Graphical abstract

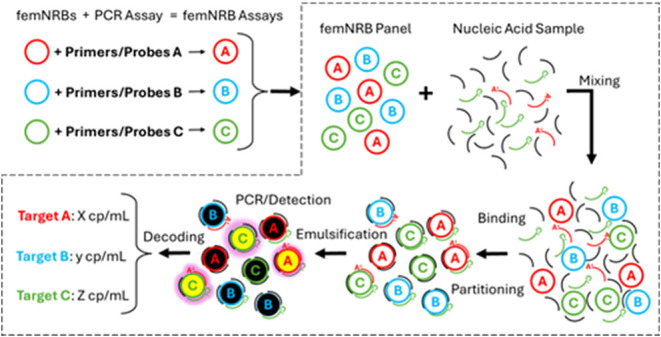

## Introduction

Accurate diagnosis is often complicated by the nonspecific nature of clinical symptoms, which may arise from a broad spectrum of underlying causes.^1–4^ Syndromic testing panels have therefore emerged as a powerful approach to accelerate diagnostic workflows and guide targeted therapeutic strategies. In particular, syndromic PCR panels enable rapid, multiplex detection of diverse molecular targets associated with overlapping symptom profiles. This strategy maximizes the diagnostic yield from a single specimen while reducing time and resource expenditure.^5,6^ Most importantly, comprehensive and timely identification of pathogens and resistance determinants enhances patient management through appropriate isolation measures and tailored treatment regimens. Such benefits are especially critical in the context of nosocomial infections and the surveillance of antimicrobial resistance.^7–9^

Despite their clinical utility, syndromic panels remain technically demanding to design and operationally complex to implement. Current assays typically yield, at best, semiquantitative results and are associated with considerable costs. Furthermore, the incorporation of additional markers into an already validated panel necessitates extensive revalidation, thereby limiting flexibility and slowing the adaptation of such systems to emerging diagnostic needs.

Multiplex PCR assays, in principle, provide sensitive and specific detection of multiple targets in a single sample. The extensive body of published research claiming advancements in the performance of multiplex PCR assays underscores as compelling evidence of the scientific importance of this topic and the considerable efforts dedicated to overcoming the associated challenges.^10–12^ To discriminate different PCR amplicons, probes are typically labeled with different fluorophores. However, this approach inherently introduces spectral overlap between fluorescence channels, which can only be partially mitigated through signal correction strategies.^13,14^ Even most advanced spectral fingerprint analysis^15^ does not compensate for the intrinsic multiplexing challenges caused by molecular competition. One approach to overcoming this challenge involves preamplification, which enhances the proportion of detectable target sequences. Subsequently, the preamplified material is distributed into discrete reaction compartments, where each target undergoes an independent detection reaction.^16^ Alternatively, microarrays^17,18^ or suspension arrays^12,19^ are employed to detect the amplification products generated in homogeneous multiplex amplification reactions. All these techniques require extensive optimization of target-specific primers and probes and face inherent intrinsic technical constraints, moreover they generally provide only semiquantitative results. Digital PCR (dPCR) addresses some of these challenges by diluting and partitioning target molecules into discrete amplification compartments, thereby eliminating competition for amplification reagents. However, the design of multiple primer-probe combinations with minimal cross-reactivity remains critical to achieving high specificity and accurate quantification.^20^

The need to minimize interassay interference has prompted the development of a class of multiplexing strategies that spatially segregate assay-specific reagents. Xie et al.^21^ present a microfluidic approach with dried, target specific primers and probes in separate chambers and a dissolvable delay valve to achieve controlled, uniform rehydration across a microwell array, enabling robust multiplex dPCR free from primer-dimer artifacts and spectral channel limits. Henley et al.^22^ employed superparamagnetic microbeads with primers coupled via thermolabile bonds to capture and purify target sequences directly from the sample. Following magnetic loading into thousands of microwells the primers have been released from the beads and amplification occurred in spatially isolated reactions, thereby eliminating crosstalk and primer dimer formation. Both approaches effectively suppress primer dimer artifacts; however, they necessitate complex microstructured substrates with well-defined nanocompartments, which imposes constraints on fabrication, assay scalability, and platform integration.

Next-Generation Sequencing (NGS) today is unparalleled in its multiplexing capacity. However, NGS workflows are hampered by extended turnaround times, high per-sample costs, and computationally intensive bioinformatic pipelines.^23^ Furthermore, NGS provides only relative abundance data; deriving absolute copy numbers typically requires external spike-in normalization or parallel dPCR controls. Consequently, for scenarios demanding rapid, absolute quantification of specific target panels, such as monitoring antibiotic resistance gene dosage or viral load, dPCR remains the superior modality, provided its multiplexing limitations can be elegantly overcome.

An overview of the methodological approaches employed for multiplexed digital PCR is provided in the Supporting Information (Table S15).

Building on magnetic Nanoreactor Beads (mNRB) we previously developed a novel digital PCR format,^24^ based on partitioning by charge mediated nucleic acid binding to highly porous hydrogel beads and subsequent formation of isolated compartments filled with amplification reagents by emulsification of the beads in oil. In this work, we present an advanced iteration of this approach, where mNRBs are individually encoded with a fluorescence code signature and reversibly loaded with target-specific primer-probe sets.

These fluorescence-encoded mNRBs (femNRBs) function as prefabricated digital single-plex PCR assays. The basic structure of such a femNRB is shown in Figure 1(A). The fluorescence coding takes advantage of a structural transformation that we observed with beads made of an agarose and chitosan mix, which results in the formation of a core–shell structure. This transformation was exploited to establish a robust coding mechanism, enabling the generation of distinct fluorescence codes. To enable primer–probe binding to specific bead codes, all femNRBs were functionalized with streptavidin, providing a simple means to immobilize target-specific primer–probe sets onto femNRBs with distinct fluorescent codes, thereby generating femNRB-associated assays (Figure 1(B)). A schematic of the partitioning, target-specific amplification, and decoding workflow with a set of three different femNRBs is shown in Figure 1(C). Following PCR amplification, the respective femNRB codes are colocalized with the PCR signal and digital PCR analysis is applied according to our previously published protocol^24^ for each femNRB independently.

**Figure 1:**
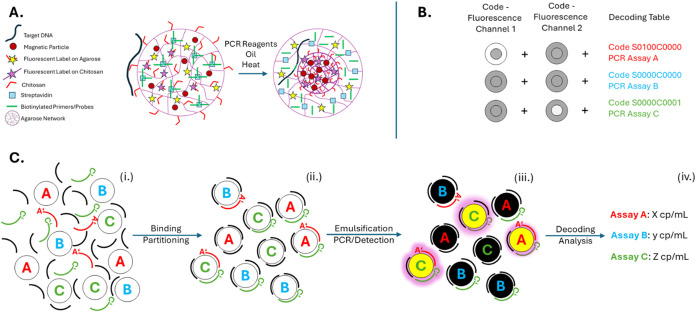
(A) Structure of femNRBs before and after thermal activation. (B) Coding principle of the shell and core compartments used to encode assay identity. (C) Schematic illustrating how three assay-specific femNRBs (A–C) are incubated with a sample containing targets for assays A (A′, red) and C (C′, green), but not B (blue). The sample also contains background DNA (black) [i]. After random distribution and binding of nucleic acids to the femNRBs [ii], the beads are emulsified, followed by PCR and fluorescence detection. Targets are only amplified in femNRBs with specific primer/probes for the respective target, shown as yellow circles [iii]. The assay performed on each bead is identified via its femNRB code, enabling separate quantification of each target [iv].

A simple workflow, as shown in Figure 2(A–E) resembles a conventional magnetic bead workflow for femNRBs that includes nucleic acid binding, purification and loading with generic PCR reagents, followed by a simple resuspension step, resulting in a femNRB suspension in oil which provides for an effective barrier between the individual reactors. During thermocycling, the agarose matrix of femNRBs liquifies and primers and probes are released. Thus, the PCR reaction within the space defined by each individual femNRB is homogeneous and occurs in isolation from other compartments. In the detection step, multiple fluorescence channels are utilized to simultaneously record the PCR signal within each femNRB and to capture a characteristic fluorescence signature. This signature functions as a specific code that identifies the PCR assay performed in the respective femNRB, as illustrated in Figure 2(F).

**Figure 2:**
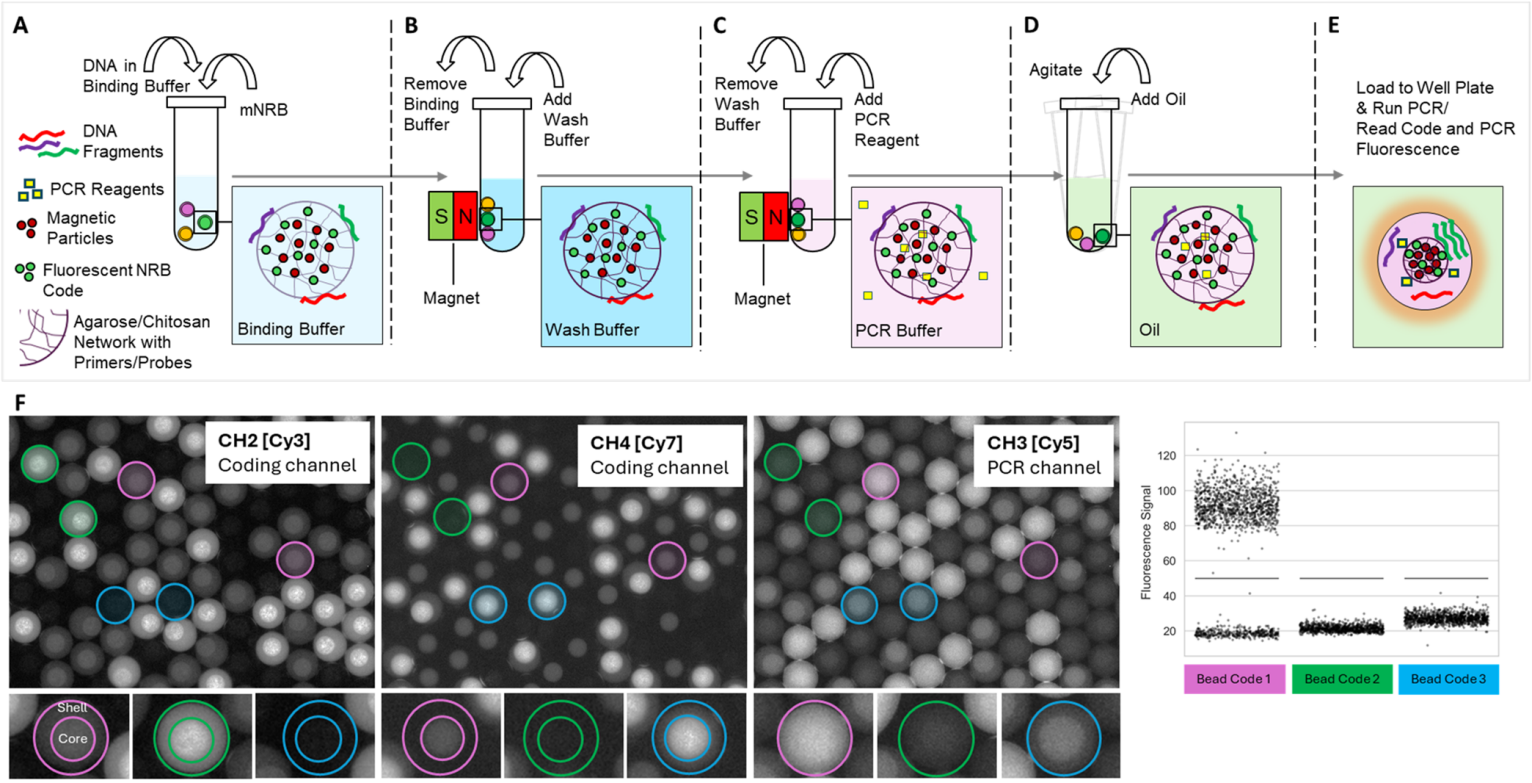
femNRB workflow with nucleic acid binding (A), washing (B), incubation with generic PCR mix (C), emulsification (D), dPCR and detection steps (E) and raw images from three fluorescence channels: CH2 (Cy3, Coding), CH3 (Cy5, PCR), and CH4 (Cy7, Coding) (F). Two channels are used for decoding, while the Cy5 (CH3) channel represents the PCR signal. For Bead Codes 2 and 3 only PCR-negative beads are shown (green and blue circles), whereas for Bead Code 1 a PCR-positive and a PCR-negative Bead are labeled (purple circles). A 1D scatter plot can be generated from the intensity values of the respective images.

We have demonstrated that these assays can be combined to generate highly adaptable multiplex PCR panels, ensuring robust detection and absolute quantification of multiple targets without detectable cross-reactivity. Our approach provides a simple and adaptable means for integrating multiple test assays into test panels, without requiring extensive optimization. Because targets contained in a sample are distributed across different beads randomly the method allows for absolute quantification of each target, independent of the number of assays included in the panel. A key prerequisite for quantification using femNRBs is the near-complete capture and consequent distribution of target molecules onto the beads. In this work we demonstrate that femNRBs fulfill this requirement (Discussion section and Supporting Information, Figure S5).

The key analytical parameters, including digital PCR measuring range can be predicted based on the total number of beads interrogated with the sample during binding and the quantity of femNRBs used per assay, irrespective of other assays present in the sample. In contrast, the detection limit for each target is defined exclusively by the number of distinct femNRB assays incorporated, similar to distributing a sample across multiple containers for different assays and the number of femNRBs utilized in the final dPCR analysis.

## Experimental Section

### femNRB Synthesis

The coding strategy relies on a multi-intensity level approach that enables robust signal discrimination through fluorescence-encoded, independently tunable femNRB compartments. For the construction of a multiplex femNRB library comprising 16 discrete codes for multiplex dPCR in this study, the fluorescence outputs of the two spatially distinct compartments formed after heating of the femNRBs in oil-the agarose shell and the chitosan core—were independently modulated by systematically varying the concentrations of their respective fluorescent labels. For each code, agarose was prepared in advance as aliquoted lyophilized gels to ensure consistency and reproducibility. The aliquots were reconstituted and melted at 99 °C for 1 h in a hybridization oven (UVP Hybridizer Oven, Analytik Jena) until complete dissolution of the agarose was achieved. All pipetting steps were performed within 30 min for consistency. The solution was then cooled to 65 °C to provide an optimal working temperature for subsequent applications. In parallel, prealiquoted lyophilized chitosan was reconstituted and equilibrated at 65 °C. A bead mixture was prepared in 50 mM MES buffer (pH 6.0) containing 500 mM NaCl, comprising unlabeled SeaKem ME Agarose (Lonza), 100 μM azide-functionalized agarose, 9.5 μM biotinylated agarose, defined amounts of Cy3-labeled agarose and Cy7-labeled agarose, resulting in a final agarose concentration of 1% (v/v).

Subsequently, different concentrations of Cy3-DBCO-functionalized chitosan, Cy7-DBCO-functionalized chitosan, and unlabeled DBCO-chitosan were incorporated, yielding a final chitosan concentration of 0.75% (v/v). The reaction mixture was incubated at 65 °C for 1 h at 500 rpm, facilitating the initiation of conjugation of azide-tagged agarose and DBCO-functionalized chitosan. The mixture was subsequently maintained at 75 °C. Magnetic particles (Cytiva), were preheated to 75 °C, subjected to ultrasonic treatment for 5 min, and added to the mixture at a final concentration of 3 mg/mL.

The reaction mixture was processed using the Blinkomat (BLINK), an automated, feedloop-controlled, pressure-driven microfluidic platform that facilitates the generation of monodisperse hydrogel droplets within a proprietary silicone oil continuous phase. The system actively monitors droplet size, homogeneity, and magnetic particle distribution through inline measurements while simultaneously removing undesired bead fractions through an integrated separation module. To maintain the liquid state of the bead mixture and prevent the sedimentation of magnetic particles, the sample was kept at 75 °C in a sample reservoir while stirring at 500 rpm.

The hydrogel droplets were collected in a tube and were incubated at 4–8 °C for at least 14 h to facilitate solidification and the formation of stable nanoreactor beads. After polymerization, the bead emulsion underwent five washing steps with Blink’s proprietary wash oil. Finally, the nanoreactor beads were washed twice in a 5-fold excess volume and resuspended in a solution containing 400 mM NaCl, 25 mM Tris–HCl, and 0.1% (v/v) PEG6000, pH 8.0 for further processing.

The bead concentration was adjusted to 700 beads/μL in the slurry. Streptavidin functionalization was accomplished by adding a 3-fold molar excess of streptavidin relative to the biotinylated agarose under agitation at 40 rpm for 30 min on a rotary wheel, followed by four washing steps with 10 mM Tris-HCl containing 0.01% (v/v) PEG6000 (pH 7.1). Bead concentration was adjusted to 500 beads/μL slurry based on femNRB counts obtained with the CASY cell counter and analyzer system (OMNI Life Science), followed by corresponding volume adjustments.

The concentrations of the fluorescently labeled components within the synthesized nanoreactor beads determine the respective code, providing distinct differentiation between labeling levels within the core (chitosan-based) and the shell (agarose-based) compartments once femNRBs are exposed to heating. The dye levels of Cy3 and Cy7 for the code set of 16 codes used within this study are summarized in Table S1 in the Supporting Information.

Each code is defined by a four-digit multilevel string for the shell (SXXXX) and a four-digit multilevel string for the core (CXXXX), where the position of the digit specifies the fluorophore type and the digit value indicates its concentration level. Positions 1 and 3 are designated for PCR-associated dyes, whereas positions 2 and 4 are allocated to coding dyes. For example, the code S0100–C0001 denotes a bead with level 1 concentration of Cy3-labeled agarose in the shell and level 1 concentration of Cy7-labeled chitosan in the core. Higher digit values correspond to higher labeling levels, thus allowing multilevel encoding beyond binary states. For the utilized multiplex set of 16 femNRB codes the levels are limited to 0, 1, 2 for coding dye 1 and 0, 1 for coding dye 2. To enable core segmentation during image analysis for each of the codes, Cy3- or Cy7-labeled chitosan may be added in lower quantities for level 0. Values are given as relative x-fold factors, where the lowest concentration in each category is defined as 1×. Higher concentrations are expressed as multiples of these references.

### PCR Assays

To test the concept of multiplexed dPCR with femNRB’s and to determine the method’s analytical performance, we used a set of previously published qPCR primer/probe oligonucleotides^25^ to detect the nine most important Gram-negative and Gram-positive bacterial pathogens causing nosocomial infections, six resistance markers relevant in this context and a human control target. All oligonucleotides were synthesized with a 5′-biotin and the primer/probe combination required for the respective assay was attached to a defined number of femNRBs with an individual fluorescence code. The femNRBs used in this work and the associated PCR assays are summarized in the following Table S2 in the Supporting Information.

Our panel is comprised of genetic markers for bacterial species identification and antimicrobial resistance detection. Species-specific markers include *aac6* for *Enterococcus faecium*, *basC* for *Acinetobacter baumannii*, *BG* for *Bacillus atrophaeus*, *cfa* for *Citrobacter freundii*, *ddl* for *Enterococcus faecalis*, *ecfX* for *Pseudomonas aeruginosa*, *gapA* for *Staphylococcus aureus*, *khe* for *Klebsiella pneumoniae*, and *sesC* for *Staphylococcus epidermidis*. The *rpp30* gene serves as a human single-copy gene for internal PCR control. The panel also includes key resistance determinants associated with clinically significant bacterial pathogens. The *mecA* gene encodes penicillin-binding protein PBP2a, which has a low affinity for β-lactam antibiotics, conferring methicillin resistance in *S. aureus* and other staphylococcal species. The *blaZ* gene encodes a class A β-lactamase, contributing to penicillin resistance by hydrolyzing β-lactam antibiotics, a common resistance mechanism in *S. aureus* and other Gram-positive bacteria. Extended-spectrum β-lactamase (ESBL)-mediated resistance is detected via *blaCTX-M1/15*, a variant of the *blaCTX-M* gene family, which hydrolyzes oxyimino-cephalosporins (e.g., cefotaxime, ceftazidime) and monobactams (aztreonam), facilitating resistance in *Enterobacteriaceae*, including *K. pneumoniae* and *C. freundii*. Carbapenem resistance is covered by markers for clinically relevant carbapenemase genes, including *blaNDM*, *blaOXA-181-like*, and *blaVIM*. The *blaNDM* (New Delhi Metallo-β-Lactamase) gene encodes a zinc-dependent metallo-β-lactamase (MBL), capable of hydrolyzing almost all β-lactams, including carbapenems, but not monobactams. This gene marker has been widely reported in *Enterobacteriaceae*, particularly *K. pneumoniae*, and is associated with multidrug resistance (MDR) profiles. The *blaOXA-181-like* gene encodes a class D carbapenemase, which exhibits weak hydrolytic activity against carbapenems but is often coexpressed with other resistance determinants, enhancing multidrug resistance in *K. pneumoniae*. Lastly, the *blaVIM* (Verona Integron-encoded Metallo-β-Lactamase) gene encodes another MBL that hydrolyzes a broad spectrum of β-lactam antibiotics, including carbapenems, and is frequently associated with mobile genetic elements, facilitating its dissemination among Gram-negative pathogens. Primer and probe for these assays have been optimized for specificity and compatibility with identical PCR annealing/elongation temperatures and PCR buffer conditions using in silico analysis followed by standard single-plex qPCR verification experiments.

### Primer and Probe Immobilization on femNRBs

For each target, biotinylated primer and probe sets were immobilized to a femNRB with an individual bead code to achieve a final in-assay concentration of 1 μM (primer) and 0.6 μM (probe), respectively. Primers and probes were mixed in a 15 mL Falcon with 3 mL loading buffer (25 mM Tris–HCl, pH 8.0, 400 mM NaCl) and incubated with 750,000 precount Nanoreactor Beads (1.5 mL) on a thermo shaker at 1000 rpm for 10 min at 30 °C. Beads were washed 1× with 3 mL loading buffer followed by three consecutive washes with 3 mL of 10 mM Tris–HCl (pH 7.1). For this the supernatant was gently removed using a magnetic tube rack and a pipet where the beads were kept on the tube wall. After the washing steps the volume in each tube is set to 1.5 mL as starting quantity. Equal volumes of Bead slurry from all individual codes were then combined forming a bead panel of 10 or 16 distinct bead codes.

### Sample Preparation/DNA Extraction

Nine different bacterial strains were cultured on Colombia blood agar plates overnight at 37 °C. DNA extraction was performed after enzymatic lysis with the Qiagen Blood&Tissue kit (Germany) according to the manufacturer’s instructions. DNA was stored in 5% Trehalose at 4 °C. Following general recommendations of standard dPCR for higher DNA amounts,^26^ DNA was fragmented by ultrasonic treatment to ensure equal distribution of DNA over all beads during binding or for generating droplets for the reference ddPCR. The resulting fragment length distribution of DNA in the respective sample has been assessed by agarose gel electrophoresis (see Figure S6 in the Supporting Information). For fragmentation 150 μL DNA solution were sonified with Branson Digital Sonifier 450D in a 1.5 mL Tube for 5× 1 s with 3 s interruption after each run with 40% amplitudes resulting in DNA fragments with an average lengths of 1200–3500 bp for bacterial DNAs and 500bp for human control DNA. DNAs were diluted with 5% Trehalose to approximately 10,000 cp/μL and for each target quantified using ddPCR (BioRad) with help of the 2× ddPCR Supermix for Probes (no dUTP) kit according to the manufacturer’s instructions with primers and probes at concentrations of 0.9 μM and 0.25 μM. The targets and species used in this work are summarized in Table S3 in the Supporting Information.

### DNA Capturing, Washing, PCR Mix Incubation, Emulsification

A total of 100,000 beads were used per experiment and capture run, corresponding to 10,000 beads per assay in the 10-plex set and 6250 beads per assay in the 16-plex set, respectively. DNA was spiked into a tube containing 50 μL capturing buffer (500 mM BTP pH 7.1), 200 μL bead solution (500 beads per μL) and 0.1% PEG with a total binding volume of 400 μL. DNA was bound to the beads for 10 min at 30 °C at 1000 rpm. After capturing the supernatant was gently removed using a magnetic tube rack and a pipet where the beads were kept on the tube wall, beads were subsequently washed once with 1 mL Bead Washing Buffer (5 mM BTP pH 7.1), and supernatant was discarded. The washed beads were then incubated for 3 min at 30 °C and 1000 rpm in 200 μL generic 1.5× PCR Mix (Blink AG, Germany) supplemented with 1 M Betain (Thermo Fisher Scientific, USA).

For the emulsification, 875 μL silicone oil including emulsifier were added. Beads were shaken for 10 s at 2100 rpm using a Blink Shaker (Blink AG, Germany). The mixture was briefly centrifuged in a table-top centrifuge, and the supernatant was discarded using a magnetic rack. This step was repeated with an additional 875 μL of silicone oil, shaken for 5 s at 2100 rpm using a Blink Shaker. After another brief centrifugation and discarding of the supernatant, 500 μL of silicone oil were added and the mixture was pipetted up and down slowly five times.

### Nanoreactor Bead PCR

3.8 μL of Nanoreactor Bead suspension were transferred to each of the six wells of the Blink X plate resulting in app. 20,000–25,000 Nanoreactor Beads loaded to each plate. In total three plates were loaded and processed on three different Blink X instruments according to the manufacturer instructions and as described elsewhere;^27^ remaining app. 25,000 femNRBs from initial input were stored as retention samples. The following PCR protocol was performed: initial denaturation at 95 °C for 60 s, 45 cycles of 95 °C for 5 s and 58 °C for 15 s.

### Performed Experiments

A set of experiments has been performed to assess performance and limitations of the new femNRB method. Different samples with varying amounts of bacterial DNA were generated, where each sample contained 100,000 cp human DNA as process control. DNA was bound to the respective multiplex panel of femNRBs using the binding and dPCR protocol. The performed experiments yielded the following data.

#### Data Set 0

Data set 0 was generated to analyze specificity of individual PCRs by using a 10-plex panel consisting of beads for detection of the nine bacterial species and rpp30 as the process and PCR control. Nine DNA samples were generated, each consisting solely of one of the bacterial DNA with nominal 20,000 copies while the other eight bacterial DNA targets were run as negative controls. Binding of DNA and dPCR were performed in three independent repeats per sample. Total number of experiments was 27. The samples used for generating data set 0 are listed in Table S4 in the Supporting Information.

#### Data Set 1

Data set 1 was generated to further analyze the specificity of individual singleplex PCRs and to determine the positive/negative threshold for each assay which were applied for all data sets. For this data set, nine different DNA samples were generated where each sample consisted solely of one of the bacterial DNA with nominal 150,000 copies while the other eight bacterial DNA targets were run as negative controls. Binding of DNA and dPCR were performed in three independent repeats per sample. Total number of experiments was 27. Samples used for generating data set 1 are shown in Table S5 in the Supporting Information.

#### Data Set 2

To analyze potential effects of the multiplex set up with all DNAs for all assays present in the sample, nine different samples were generated each containing a mixture of nine bacterial DNAs where eight DNAs were adjusted to 150,000 cp/sample and one bacterial DNA was used at 20,000 cp/sample. DNA samples were analyzed using the 10-plex panel. Binding of DNA and dPCR were performed in three independent repeats per sample. Total number of experiments was 27. Samples used to generate data set 2 are listed in Table S6

#### Data Set 3

For further analyzing the precision of the method, a sample was generated containing a mixture of nine bacterial DNAs where all DNAs were adjusted to 20,000 cp/sample, Binding was performed in four independent repeats, Beads of one binding step were applied to three experiments, total number of experiments was 12, for analyzing the samples, the 10-plex panel was used. The samples used to generate data set 3 are listed in Table S7 in the Supporting Information.

#### Data Set 4

To further determine specificity of the femNRBs nine samples were generated containing eight bacterial DNAs each with 150,000 cp/sample and one bacterial DNA not present (negative control), Binding of DNA and dPCR were performed in three independent repeats per sample, Total number of experiments was 27. The samples used for this set of data are shown in Table S8 in the Supporting Information.

#### Data Set 5

Another experiment was run to analyze the linearity of the method at least for one example target in the multiplex setup. Six different samples were generated where each sample contained eight bacterial DNAs with a nominal DNA concentration of 150,000 cp/sample and the bacterial DNA containing the gapA target at varying concentrations from 2000 to 250,000 cp/sample, respectively. These gapA DNA concentrations were also tested with femNRBs for gapA in a singleplex assay. Binding of DNA and dPCR were performed in three independent repeats per sample. Total number of experiments was 18. The multiplex experiment with varying concentrations of one out of ten targets was repeated with ddl and sesC. The sample composition used for generating this set of data is shown in Table S9 in the Supporting Information.

#### Data Set 6

The 10-plex panel was expanded with six resistance gene markers by adding six additional femNRBs with different bead codes with specific primers and probes. Four different samples were prepared containing first individual DNAs for *A. baumanii*, *S. aureus* and *K. pneumoniae* and finally a mixture of DNAs from all three species. The samples used for generating data set 6 are shown in Table S10 in the Supporting Information.

### Bead Decoding by Robust Clustering

Fluorescence images were processed by applying a segmentation algorithm allowing for detection of beads and providing gray value information for local bead background, shell and core signal. The algorithm also recognizes imaging artifacts and excludes beads from analysis which do not fulfill quality criteria like shape, diameter and gray value distribution. For each bead contrast values were calculated by subtracting background intensity from the mean shell signal and by computing the difference between core and shell signals, with all values normalized by exposure time and gain applied during imaging. Two encoding channels (CH2, CH4) with the two different compartments (shell and core) were used for bead coding, resulting in four numerical features per bead.

Bead classification was performed using a Gaussian Mixture Model (GMM) with full covariance matrices, which enables flexible modeling of elliptical clusters with varying sizes and variances. The number of clusters was fixed according to the used bead code library definitions for the 10- and 16-plex multiplex panels. Model parameters were estimated using the Expectation–Maximization (EM) algorithm, and soft clustering provided posterior probabilities for each bead, facilitating uncertainty assessment in ambiguous cases.

To ensure robust initialization, GMM parameters were seeded by agglomerative hierarchical clustering applied to a reduced, log-transformed data set. Noise resilience was further improved by adapting the Expectation-Maximization (EM) algorithm to include a Minimum Covariance Determinant (MCD) estimator^28,29^ for cluster means and covariances.

Postprocessing steps included filtering of low-confidence assignments using Mahalanobis distance-based confidence ellipsoids and exclusion of beads located in intercluster boundary regions. Bead type validity was further assessed by requiring a minimum number of valid beads per cluster.

To further assess cluster separation, the Bhattacharyya coefficient^30^ was calculated between cluster distributions, where smaller values reflect greater statistical distinctness. For Gaussian-distributed clusters, this metric provides a principled estimate of intercluster separability by quantifying the degree of distributional overlap and an upper bound on the classification error.

### dPCR Analysis

For each target and bead code the respective target concentration of detected DNA was calculated based on the following formula.

1 $$C_{s}=\frac{N_{B}}{V_{s}}\cdot -\ln\left(\frac{N_{neg}}{N}\right)=\frac{N_{B}}{V_{s}}\cdot \lambda$$

The mean number of targets per bead (λ) is calculated using the same Poisson statistics as for conventional digital assays^31^ with *N*_neg_ as the number of PCR negative beads and *N* the total number of beads detected. Since binding is used as the method of compartmentalization no information on the bead volume is required. Instead, the total number of beads (*N*_B_) applied to (*V*_S_) and multiplied with the λ-value.

### femNRB Binding Kinetics and Capture Efficiency

To characterize the capture efficiency and binding kinetics of the femNRBs, supernatants from binding reactions were collected at multiple time points (0, 1, 2, 4, 8, 10, and 16 min) for three different fragment sizes (∼400 bp, 1300 bp and >20000 bp) of *S. aureus* DNA and in two concentrations (*N*_1_ = 4.48 × 10^4^ copies, λ = 2.5 and *N*_2_ = 4.48 × 10^6^ copies, λ = 250) incubated with 20,000 femNRBs in an 80 μL binding mixture (250 beads/μL). The selected concentrations were chosen to span the range of DNA inputs used throughout the present study. Aliquots of the supernatants were analyzed using ddPCR Supermix for Probes (No dUTP, Bio-Rad) on the QX200 Droplet Digital PCR system according to the manufacturer’s instructions.

### Testing of Potential Primer Diffusion

To assess potential primer diffusion between bead types, two distinct bead codes (A and B) were generated. Bead code A carried the immobilized probe alongside the forward and reverse primers targeting rpp30, whereas bead code B carried only the probe and forward primer for the same target. The two bead types were combined at a 1:1 ratio, and a nominal of 50,000 copies of human genomic DNA were bound to a total of 25,000 beads consisting of the bead code mix. dPCR was subsequently performed, and target copy numbers for each bead code were derived from the calculated lambda values.

### Sequencing

Whole-genome sequencing of all strains was performed using the Oxford Nanopore Technologies (ONT) MinION platform to confirm species identity and characterize resistance gene profiles. Genomic DNA was extracted using the NucleoSpin Microbial DNA Kit (Macherey-Nagel, Düren, Germany) with minor protocol modifications. Bacterial isolates were cultured overnight on Columbia blood agar (Becton Dickinson, Heidelberg, Germany), and biomass was collected using a full inoculation loop. Cells were suspended in 500 μL PBS (pH 7.4), pelleted by centrifugation, and resuspended in 100 μL buffer BE. Mechanical lysis was achieved using a Bead Beater (Biozym, Hessisch Oldendorf, Germany) for 5 min at maximum speed. Proteinase K digestion was followed by heat inactivation at 70 °C for 5 min. RNase A (100 mg/mL; Sigma-Aldrich, Steinheim, Germany) was then added and incubated at 37 °C for 5 min. DNA was purified and eluted in 70 μL of nuclease-free water (Carl Roth, Karlsruhe, Germany).

Library preparation was conducted using the SQK-NBD114.24 Native Barcoding Kit (Oxford Nanopore Technologies), and sequencing was performed exclusively on R10.4.1 flow cells (FLO-MIN114). DNA was size-selected using AMPure XP beads (Beckman Coulter, Krefeld, Germany) at a 1:1 ratio to enrich for high-molecular-weight fragments. Sequencing runs were executed for 72 h using MinKNOW (v23), and raw signal data were recorded in POD5 format.

## Results

We have developed and synthesized a set of 16 different femNRB and used this set to evaluate the performance and limitations of the novel femNRB-based multiplex digital PCR analysis format. A structured series of experiments was conducted using a 10-plex bead panel targeting nine bacterial DNA target sequences alongside rpp30 as a process and PCR control. All samples included 100,000 copies of human DNA prequantified by ddPCR to monitor assay integrity. The experimental design encompassed both singleplex and multiplex configurations, with varying target concentrations and sample compositions. Analytical specificity, sensitivity, reproducibility, and dynamic range were systematically assessed across multiple data sets. These included single-target samples for threshold calibration, mixed samples with defined copy number ratios to probe detection limits, and complex DNA backgrounds to evaluate cross-reactivity and quantification accuracy. Additionally, the panel was expanded to incorporate highly relevant antimicrobial resistance gene markers, enabling preliminary validation of broader diagnostic applicability. The following sections detail the experimental conditions, sample compositions, and analytical outcomes across seven distinct data sets, providing a comprehensive characterization of the femNRB platform’s capabilities.

### femNRB Library

Sixteen different femNRB species each containing a different fluorescence code were produced. To realize the fluorescence code we employed the fact that the mixture of chitosan and agarose that constitutes the femNRBs undergoes a notable structural change under PCR buffer conditions when subjected to heating, resulting in the formation of a core consisting primarily of chitosan and a shell consisting primarily of agarose (see Figure 1(A)). This configuration offers two compartments for the encoding of beads with fluorescent dyes, which can be spatially differentiated by means of fluorescence imaging. This presents a novel avenue for the implementation of combinatorial codes with a limited number of fluorescence channels and intensities. Figure 3 illustrates the approach and the composition of the 16 femNRB with their respective codes used in this work.

**Figure 3:**
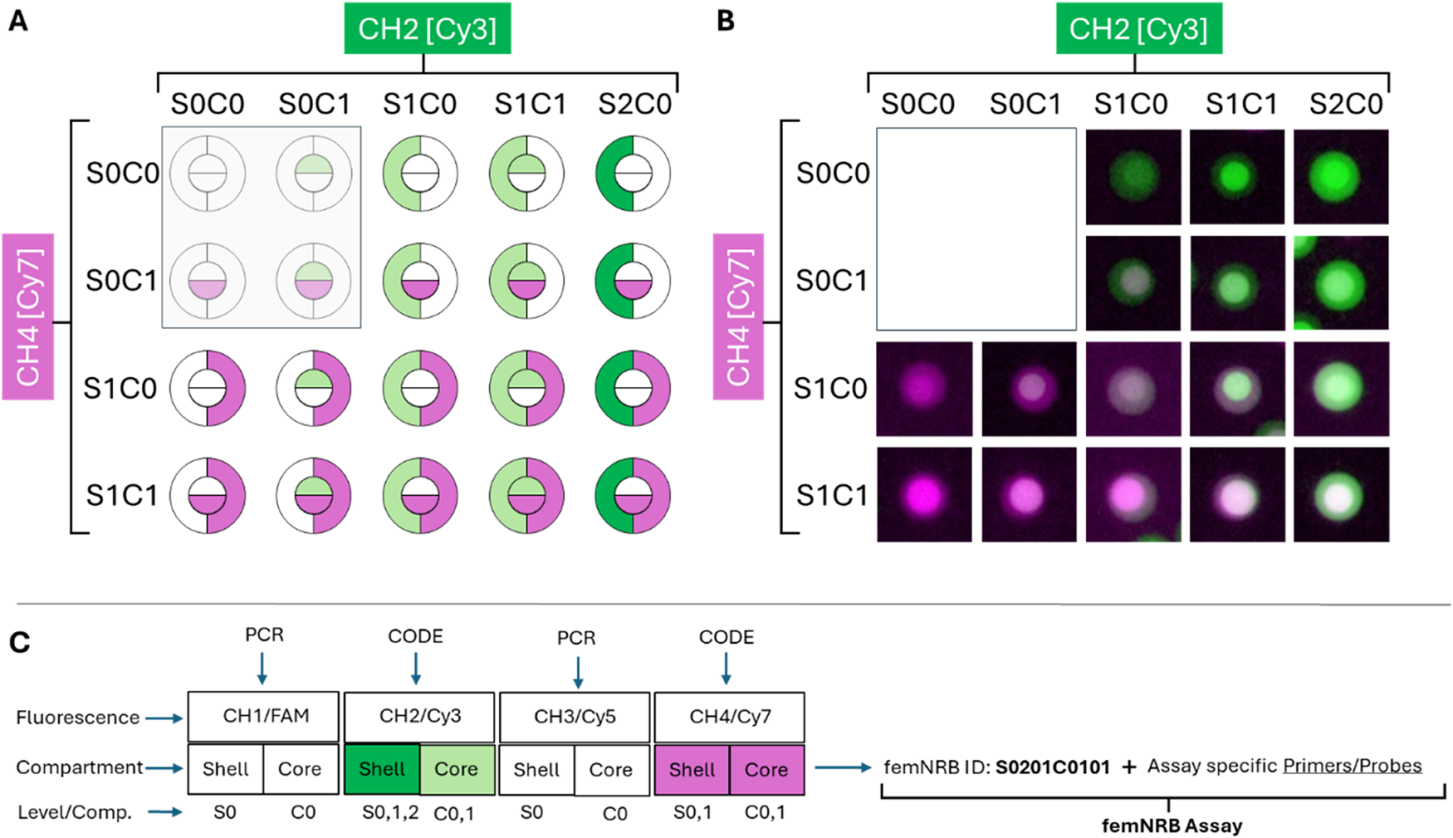
(A) Fluorescence Coding Scheme, (B) Fluorescent image overlays of femNRBs for each code used in the 16-plex panel after heating showing distinctive shell and core fluorescent signal, (C) schematic for coding scheme for individual bead codes.

Code specific fluorescence has been detected through the Cy3 and Cy7 fluorescence channels of the BLINK X system, whereas the FAM and Cy5 channels remained available for PCR signal detection. To facilitate the detection of the beads by automatic pattern recognition algorithms of the BLINK X software, it was imperative to ensure that the shell area of all beads produced was fluorescence labeled in one of the two coding channels. Thus, to generate 16 codes that meet this requirement, it was necessary to use intensity level differences as a distinguishing feature for some of the bead codes. A software algorithm was developed to correctly identify the bead codes. A description of the data analysis workflow is shown in Figure S1 and a flowchart of employed bead decoding procedures in Figure S2 in the Supporting Information.

The median Bhattacharyya coefficient per test of all bead decodings performed is 1.47 × 10^–08^. This value specifies an upper limit for the risk of misclassification. The probability of 1.47 × 10^–08^ corresponds to one incorrectly classified bead out of a total number of 67,970,000 beads. Exemplary scatter plots that visually illustrate the separability of 10 and 16 codes are shown in the Supporting Information, Figure S3. Median value, interquartile range and max value of Bhattacharyya coefficients across experiments, the proportion of valid beads and the final valid bead count per code are reported in the Supporting Information, Table S11.

### 10-plex PCR Assay

Ten different femNRBs have been bound with Primers and Probes for ten different assays targeting nine bacterial specific markers (AAC6, basC, BG, cfa, ddl, ecfX, gapA, khe, sesC) and rpp30, a human genomic marker as process control. A library comprised of the ten Primer/Probe bound femNRBS has been generated by mixing equal numbers of individual femNRBs. The generated femNRB library has been incubated with each target individually at two different concentrations (set up for data set 0 and data set 1). Singleplex ddPCR (Bio-Rad) was used as the reference method to determine the absolute concentration of the respective target in each sample. A comparison of the copy number results from data set 0 and data set 1 with the respective reference values shows a high level of agreement between the absolute results obtained with mNRBs and the ddPCR reference method with the mean deviations over all ten targets between both methods of 97.4% (data set 0) and 101% (data set 1), respectively. Based on this data we calculated the recovery for each target under the respective experimental conditions and summarized the data in Table 1.

**Table 1:**
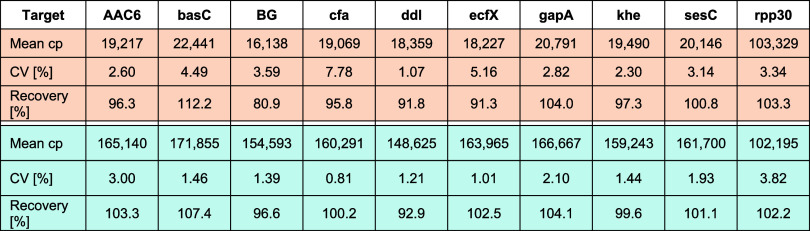
Summary of Recovery Data for Data Set 0 (Orange) and Data Set 1 (Blue)

### 10-plex Assay Specificity

Results from data sets 0, 1, and 4 were used to determine specificity of the multiplex panel following CLSI EP17-A2 (nonparametric approach) for calculating a limit of blank (LoB). In total 81 experiments (BLINK X plates) were considered as independent runs and the number of false positive beads per run was analyzed. For each experiment, a lambda value was calculated based on the ratio between false negative beads and valid beads. Results were sorted in ascending order of lambda values. In total five runs have been found with exactly one false positive bead each where the highest lambda value was 0.000058 or one false positive bead out of 17,241 valid beads resulting in a LoB of 0.000058. By aiming for 100% specificity, a lambda of 0.000067 (1 out of 15,000 beads) as LoB would result in 100% specificity with a 95% confidence interval of [95.55, 100.0]. For a complete plate with 20,000 beads this would lead to a cutoff of 1.34 copies per sample as LoB.

### Paired T-Test for Single DNA and Multiplex DNA Samples

For data set 2 in total nine different mixtures were tested, where each mixture contained eight of the bacterial DNAs at a high-level between 128,474 cp/sample and 173,275 cp/sample and one bacterial target ranging from 16,544 cp/sample and 21,616 cp/sample. A paired sample *t* test was performed comparing each high-level DNA target from data set 2 with the results of data set 1 where only one bacterial DNA was present at a high level. A *p*-value of 0.577 was calculated, indicating no statistically significant differences between the two experimental setups with one bacterial target and with all targets present.

Another *t* test was performed using the low-level target results from data set 2 and comparing it with data set 3 where all targets were present at the lower-level ranging between 18,631 cp/sample and 22,700 cp/sample. Paired sample *t* test resulted in a *p*-value of 0.156.

### Precision

Precision was estimated at 20,000 and 150,000 input copies for nine targets. We used data set 3 to calculate precision measures for the low DNA sample and data sets 2, 4, and 5 for the high DNA samples. All four data sets used represent multiplex conditions.

Formula [Disp-formula eq2] was used to calculate the within-data set precision expressed as standard deviation. Since different aliquots of DNA were used for the various data sets, an overall precision calculation is not appropriate. Instead, the variances of the different data sets were determined separately and averaged.

2 $$s=\sqrt{\frac{\sum_{d}^{D}\sum_{n}^{N}{\left(x_{d,n}-{\mathrm{x̅}}_{d}\right)}^{2}}{D\cdot \left(N-1\right)}}$$

*D* is the number of data sets and *N* is the number of replicates. *X*_*d*,*n*_ represents the lambda value of replicate *n* in data set *d*. *x̅_d_* is the mean lambda value of all replicates in data set *d*.

Coefficients of variation for the low DNA sample range between 4.78% and 9.69% while coefficient of variation results for the high DNA sample range between 3.13% and 5.41%, respectively. The results confirm the expected outcome that achievable precision is better at 150,000 input copies than at 2,000 input copies which corresponds to the inherent statistical uncertainty due to Poisson distribution of copies in the sample volume and the Poisson distribution of copies in the beads.

Calculated precision measures involve not only the variation between binding repeats but also variance components for different instruments and different experiment days. Considering this fact, CV values below 10% are in line with previously published data for Nanoreactor Beads.^24^ Tables S13 and S14 in the Supporting Information summarize the precision results for low target concentrations (data set 3) and high target concentrations (data sets 2, 4, and 5), respectively.

### Linearity and Quantification

Six different samples were generated, where each sample contained eight bacterial genomic DNA samples with nominal DNA concentrations ranging from 150,000 to 170,000 cp/sample depending on the target and the gapA target DNA at varying concentrations from 2000 to 250,000 cp/sample. These gapA DNA concentrations were also tested with femNRBs for gapA in a singleplex assay. Figure 4 provides a direct comparison between the singleplex and multiplex results.

**Figure 4:**
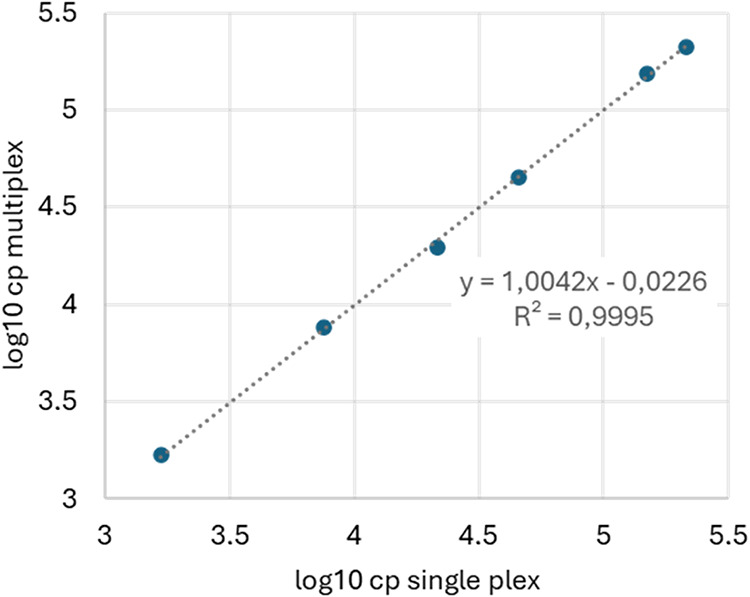
Correlation of gapA measurements across the dilution series for singleplex and multiplex assay formats.

We analyzed the correlation between the gapA results for the different gapA target concentrations from data set 5 and found perfect linearity and correlation between samples containing all 9 bacterial targets and samples with gapA target only. The multiplex experiment was repeated with ddl and sesC targets which were present at six different concentrations (see Figure 5(A–C)).

**Figure 5:**
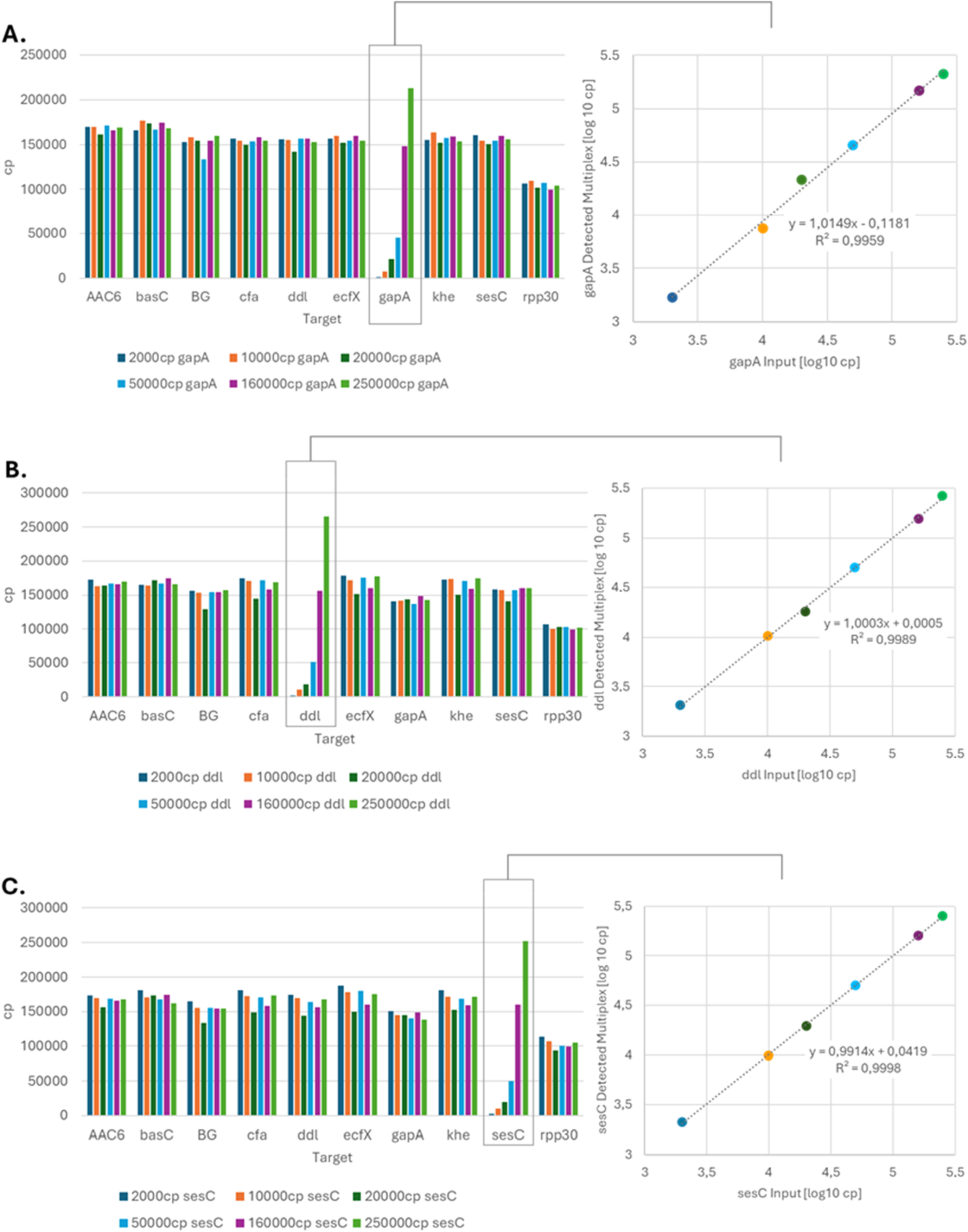
(A–C) Results of the gapA, ddl, and sesC target dilution series in the multiplex setting with eight bacterial targets, each present at approximately 160,000 cp per sample, and 100,000 cp per sample of the rpp30 target as a positive control.

A paired *t* test analysis for the gapA target run as singleplex and as multiplex was performed. A *p*-value of 0.701 was calculated for comparing the gapA results from the experiment where only the gapA DNA was present with the experiments where gapA DNA and all other eight bacterial targets were present.

### 16-plex Panel Expansion

The experiments with the 10-plex panel have shown that the individual test assays allowed excellent quantification for each individual target and that no cross-reactivity between the individual PCR assays in the panel was observed. To be able to determine resistance markers in addition to the species markers, we expanded the panel by six additional femNRBs and PCR assays and carried out tests with DNA isolates of individual bacterial strains and a mixture of DNA isolates from three different bacterial strains. As a reference, we performed all PCR assays included in the panel as singleplex ddPCR assays with the same samples (Figure 6).

**Figure 6:**
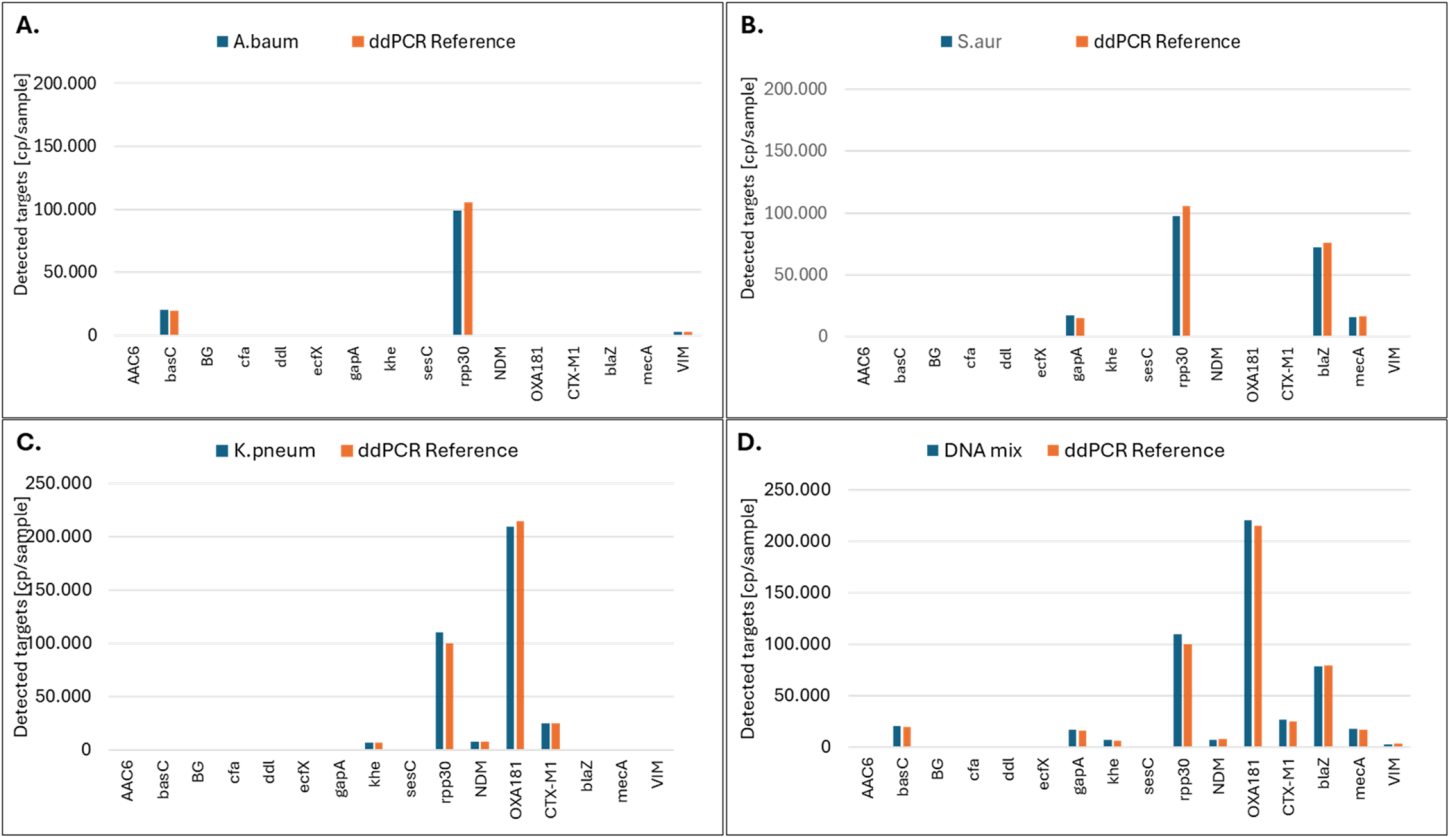
Test results obtained with the 16-plex panel using three different bacterial strains and a bacterial mixture. (A): Detection profile for *A. baumanii*, (B): Detection profile for *S. aureus*, (C): Detection profile for *K. pneumoniae*, and (D): Detection profile for a mixture of samples. In each subgraph, the panel result is shown in blue and the reference result determined by singleplex ddPCR is shown in orange. As quantitative control, human genomic DNA at a nominal concentration of 100,000 cp/sample was used in all experiments. Copy numbers for singleplex ddPCR reference (orange) and for 16 plex femNRB (blue) are shown for single DNA-Isolates present in the sample (A–C) and for a mixed sample containing three different DNA isolates from *A. baumanii*, *S. aureus* and *K. pneumoniae* (D).

Comparison of the results showed excellent correlation of *R* ^2^ = 0.9974 for all targets between the data obtained with the 16-plex femNRB panel using a DNA mixture and the individually performed singleplex ddPCR with single DNAs present in the reaction, which is in line with the performance of the 10-plex panel.

In all experiments conducted, the λ value was calculated according to Formula [Disp-formula eq1] described in the Experimental Section. As this calculation assumes absolute quantitative capture of targets onto the femNRBs, capture efficiency and binding kinetics were evaluated. Under the binding conditions used in this study (10 min, 30 °C, 1000 rpm, 250 beads/μL), capture was shown to be ≥99% within 10 min for two concentrations of bacterial *S. aureus* DNA tested with three different fragment lengths (see Figure S5 and Table S12 in the Supporting Information). Binding kinetics depend on DNA fragment length and DNA amount where minimum time to reach 99% binding (*t*_99_) was 2.03 min for λ = 2.5 and <400 bp DNA, maximum time was 8.77 min for λ = 250 and unfragmented DNA (>20,000 bp). This is in line with previous work that demonstrated ≥99% capture within 10 min, under similar binding conditions (10 min, 30 °C, 1000 rpm, 139 beads/μL) using sonicated DNA at concentrations exceeding the range of dPCR.^24^ Thus, bead-bound target concentration equals the original sample concentration, enabling absolute quantification on femNRBs. This observation is also supported by comparison of absolute copy number from ddPCR and femNRBs in data sets 0–4 where following recoveries were calculated over all 10 targets: 97.4% (data set 0), 101.0% (data set 1), 101.4% (data set 2), 99.5% (data set 3), 100.4% (data set 4). Average recovery over these data sets was 99.94% with 97.8% and 100.03% for lower and upper 95% confidence intervals, suggesting app. 100% recovery rate for Nanoreactor Beads in performed experiments. Unfragmented DNA may require longer binding times to achieve >99% binding.

To evaluate potential primer diffusion between bead codes, two codes were generated: code A containing the probe, forward primer, and reverse primer for *rpp30*, and code B containing only the probe and forward primer. DNA was bound to the mixed bead population and analyzed by dPCR. Code A showed a lambda value of 2.24, corresponding to 55,934 *rpp30* copies during the binding step. No positive nanoreactor beads were detected for code B, demonstrating an absence of detectable primer diffusion under the tested conditions.

For each strain the copy number ratio (S:R) of species (S) and resistance (R) markers from femNRB dPCR results were calculated and referenced with ONT sequencing and assignment to respective chromosomal or plasmid contigs of each strain. In *K. pneumoniae*, NDM resistance target was localized on a large low-copy plasmid (298,421 bp) with a ratio of 1:1 in dPCR. The OXA-181/232 target was detected on a small high-copy plasmid (6,140 bp) with a ratio of 1:33 in dPCR. The CTX-M1 target was found both on the chromosome and on a large low-copy plasmid (69,623 bp) with a total S:R ratio of 1:4 in dPCR. In *S. aureus*, the chromosomal species marker gapA (2,820,463 bp) served as reference. The blaZ target was identified on a plasmid (20,654 bp) and showed a S:R of 1:5 in dPCR, indicating higher relative abundance of the resistance target compared to the chromosomal marker. The mecA target was located on the chromosome (2,820,463 bp) with a S:R ratio of 1:1 in dPCR. In *A. baumannii* the VIM target was localized on a large low-copy plasmid (46,757 bp) with a S:R ratio of 8:1 in dPCR. No additional resistance genes were identified in this strain. A summary of the data is provided in Table 2.

**Table 2:** Respective Ratio of Species Marker (S) and Resistance Marker (R) Determined for Each Strain by femNRB dPCR; Location and Size of Each Target Obtained by ONT Sequencing

	species marker		resistance marker								
strain	target	cp/target; location (size)	target	dPCR ratio (S:R)	cp/target; location (size)	gene	dPCR ratio (S:R)	cp/target; location (size)	gene	dPCR ratio (S:R)	cp/target; location (size)
*K. pneumoniae*	*khe*	6,649; Chromo-some (5,355,385 bp)	NDM	1:1	6,821; Plasmid (298,421 bp)	*OXA-181*	1:33	220,448; Plasmid (6140 bp)	CTX-M1	1:4	26,211; Chromo-some (5,355,385 bp), Plasmid (69,623 bp)
*S. aureus*	*gapA*	16,559; Chromo-some (2,820,463 bp)	*blaZ*	1:5	78,731; Plasmid (20,654 bp)	*mecA*	1:1	17,133 Chromo-some (2,820,463 bp)			
*A. baumannii*	*basC*	19,807; Chromo-some (4,112,957 bp)	*VIM*	8:1	2,434; Plasmid (46,757 bp)						

## Discussion

We have introduced a novel class of magnetic nanoreactor beads (femNRBs) that integrate fluorescence encoding with target-specific primers and probes, forming discrete assay compartments for digital amplification and detection. These beads allow for the flexible assembly of digital PCR (dPCR) panels from existing singleplex qPCR assays, without requiring further optimization to mitigate cross-reactivity between the respective PCR reactions. Each femNRB functions as an autonomous PCR amplification compartment, enabling precise quantification of individual targets within a multiplexed format. Our method overcomes current limitations in dPCR multiplexing like signal cross-talk, specificity, sensitivity and primer and probe design challenges.

Direct comparison of data obtained with femNRB panel assays with conventional droplet digital PCR (ddPCR) controls reveals excellent concordance in target concentration measurements, underscoring the analytical robustness of the femNRB platform. We have shown independent quantitation of 16 targets in a single reaction without detectable cross-reactivity. Statistical analysis confirms the absence of any significant bias between multiplexed, singleplex and ddPCR reference data sets, with coefficients of variation (CV) values averaging 4.01% for high and 7.74% for low DNA input concentrations for the femNRB method. These variations are primarily attributable to pipetting inconsistencies during the DNA binding step. Bead-coded multiplex digital quantification enables parallel measurement of multiple targets while preserving Poisson-based digital PCR principles. However, multiplexing entails a predictable statistical trade-off: as the number of targets and bead codes increases, the number of partitions available per target decreases, thereby narrowing the quantitative range for each analyte. At a fixed total bead count, each target is quantified only within its bead subset. With more bead codes, fewer partitions per code remain, and the dynamic range of each target contracts. Figure S4 in the Supporting Information shows different multiplexing levels and corresponding lower and upper measurement limits together with their expected CV using the standard expression for λ-estimate variance in digital PCR.^32^ The lower range is limited by the LOD, which increases with multiplexing because target molecules are distributed across multiple bead codes. Assuming a zero limit of blank, the theoretical minimum LOD is three molecules per reaction (95% probability of ≥ 1 positive partition). When multiplexing n-fold, the effective LOD scales approximately proportionally with n. Like in conventional dPCR and applying Poisson statistics, the upper limit of quantification is directly affected by the total number of available partitions (beads) per target or bead code and therefore also depends on the level of multiplexing when assuming a defined total amount of beads for all targets. As the Nanoreactor Bead workflow allows for utilization of large sample/eluate volumes theoretical LOD limitations can be compensated. For samples exceeding the digital measurement range, the reduced ULOQ in high-plex panels can be addressed by increasing the total number of beads or determining target copy numbers based on the cq value for each bead code as shown previously.^33^

The underlying image analysis algorithm reliably excludes artifactual signals and merged bead events, preventing overestimation of target abundance. Specificity assessments under multiplex stress conditions revealed no false-positive signal propagation across targets with LoB comparable to standard dPCR, further validating the system’s orthogonality. On average, 10.5% of the femNRBs loaded onto the plate in the 10-plex assays and 10.8% in the 16-plex assays were classified as invalid by the software and excluded from downstream analysis. In addition, workflow-related losses during pipetting, washing, and transfer steps accounted for approximately 5–15% of the initial femNRB input. The mean number of valid beads analyzed per plate and experiment was 18,990 for the 10-plex assays and 19,664 for the 16-plex assays equally distributed over bead codes.

Notably, femNRBs when using purified nucleic acids exhibit near-complete target recovery and uniform distribution of bound nucleic acids across the bead population, supporting consistent assay performance. Binding kinetics and overall capture efficiency depend on DNA fragment length as well as on concentration and may be altered by matrix effects when nucleic acids are bound to femNRBs directly from a lysed sample.

We also show that reagents originally developed for qPCR can be directly repurposed within the femNRB format, significantly reducing assay development timelines for syndromic panels. We anticipate that this compatibility will streamline regulatory validation workflows for in vitro diagnostic (IVD) applications and revalidation efforts for expansion of existing assay panels will be facilitated by simply adding new femNRBs. The ability of cross-talk free multiplexing offers a transformative advantage, particularly in contexts such as emerging pathogen surveillance and rapid detection of new resistance patterns. For the three bacterial strains tested with the 16-plex panel the determined species to resistance ratios provided a coherent view of resistance gene carriage and likely dosage effects which can be supported by sequencing data and the respective locations of the individual targets. In *K. pneumoniae*, NDM-1 was found to reside on a large, low-copy plasmid which is consistent with a single-copy carriage per chromosome equivalent to a low-copy backbone. This agrees with the broad observation that NDM determinants circulate on low-to-moderate copy, conjugative plasmids (e.g., Inc.F/Inc.C/Inc.X3) in *Enterobacterales*.^34,35^ By contrast, OXA181/232 was found on a 6-kb ColKP3/ColE-type high-copy plasmid. The tight association of OXA-181/232 with 6-kb ColKP3 plasmids is now well documented in clinical *Klebsiella* lineages and is frequently nonconjugative but highly stable, consistent with elevated copy numbers.^36,37^ The gene CTX-M-15 in the same isolate appeared both chromosomally and on a large, low-copy plasmid, explaining the 1:4 species to resistance ratio. Dual localization of CTX-M-15 is increasingly recognized: while plasmid carriage (often Inc.F-type) remains common, chromosomal integration events are reported in multiple *Klebsiella* lineages and can stabilize ESBL determinants in the absence of plasmid selection. The observed mixed localization therefore fits with published patterns of CTX-M-15 mobility between plasmid and chromosome.^38^ In *S. aureus*, the *blaZ* gene was located on a plasmid of approximately 20 kb, whereas *gapA* and *mecA* are chromosomally located. Digital PCR analysis revealed a S:R ratio of 1:5 for *blaZ*, indicating a higher relative abundance of the resistance target compared with the chromosomal marker. The size and structure of the *blaZ*-bearing element correspond to the small to medium-sized β-lactamase plasmids (≈20–30 kb) commonly associated with *blaZ* carriage in *S. aureus*, frequently involving Tn552-like transposons.^39^ The S:R ratio of 1:1 for *mecA* and its chromosomal location corresponds to its well-established single-copy integration within the SCCmec element.^40^ The co-occurrence of plasmid-borne *blaZ* and chromosomal *mecA* reflects a resistance architecture typical of methicillin-resistant *S. aureus* (MRSA) strains, combining β-lactamase production and altered PBP2a-mediated resistance mechanisms. In *A. baumannii*, VIM was on a large, low-copy plasmid with a S:R ratio of 8:1 with substantially fewer resistance targets per chromosomal equivalent than expected for a uniformly single-copy plasmid. Several (nonexclusive) scenarios align with this: (i) subpopulation carriage (plasmid present in a minority of cells), (ii) unstable/segregational loss of a low-copy plasmid, (iii) assay-level under-quantification (probe/primer efficiency), or (iv) structural variation (e.g., integron context or partial deletions) that reduces effective target copies. Notably, VIM is commonly embedded in class 1 integrons borne by plasmids in nonfermenters (including *Acinetobacter*), which can facilitate mosaic structures and variable stability -consistent with the depressed effective copy signal.^41^ Overall, the copy number variation ratio between species and resistance markers could serve as a characteristic fingerprint for strain identification which can be easily determined by the presented method. Similar to other dPCR methods, copy number variations could be altered by the fragmentation degree of DNA especially if multicopy resistance markers are present on the same plasmid.

As femNRBs provide a scalable fluorescence encoding strategy, the coding space can be readily expanded, enabling the parallel development of increasingly complex test panels without compromising assay independence or detection fidelity. This modularity supports rapid adaptation to evolving diagnostic needs. Beyond IVD, the femNRB platform opens new avenues in fields requiring high-throughput, quantitative nucleic acid analysis. Potential applications include food and beverage safety testing, plant genotyping, wastewater surveillance, and environmental monitoring. The scalable nature of the technology, combined with its compatibility with standard laboratory infrastructure and point-of-care formats, positions femNRBs as a versatile solution for decentralized molecular diagnostics.

## Supplementary Material



## Notes

Comprehensive overview of the materials, samples, and analytical procedures used in this study: Table S1 lists the fluorescent dye labels and relative dye levels of the 16-plex femNRB set, sorted by Cy3/Cy7 core and shell intensities, while Table S2 links each femNRB to its corresponding PCR assay, Table S3 summarizes all genetic material used in this work, Tables S4–S10 detail the sample compositions for data sets 0–6, including ddPCR-based reference samples and a mixed sample, Table S11 reports Bhattacharyya coefficients, bead validity, and bead counts across all experiments; the figures illustrate key analytical workflows and performance characteristics: Figure S1 shows the data-analysis pipeline, Figure S2 the bead-decoding procedure, and Figure S3 the distribution of decoding features for experiments with 10 and 16 bead types, Figure S4 presents the dPCR measuring range at different multiplex levels, and Figure S5 shows binding kinetics of *S. aureus* DNA across fragment lengths and concentrations, Table S12 provides estimated *t*-values with confidence intervals and *R* ^2^ values, Figure S6 displays agarose gel electrophoresis results of ultrasonically fragmented DNA, Tables S13 and S14 summarize precision results for low and high DNA inputs, Table S15 offers a comparative overview of multiplexing strategies in digital PCR (PDF)
