# Supplementary material for: Quantitative Multiplex Digital PCR with Fluorescence-Encoded Nanoreactor Beads
Source: Anal Chem. 2026 May 18;98(21):15479–92. doi: 10.1021/acs.analchem.6c00259 (PMC13234817; doi:10.1021/acs.analchem.6c00259)
Supplement: Supplementary file 1 [file ac6c00259_si_001.pdf]

SUPPORTING INFORMATION TO:

"**Quantitative Multiplex Digital PCR with Fluorescence Encoded Nanoreactor Beads**"

Stephan Hubold[1], Lea Kanitz[1], Oliver Lemuth[1], Ines Engelmann[1], Susanne Toepfer[1], Susanne Brandes[1], Theresa Liebe[1], Friederike Fritsch[1], Sascha Braun[2], Martin Reinicke[2], Ralf Ehricht[2] and Eugen Ermantraut[1*]

[1] BLINK AG, Bruesseler Strasse 20, 07747 Jena, Germany

[2] Leibniz Institute of Photonic Technology, Member of the Research Alliance "Leibniz Health Technologies" and the Leibniz Centre for Photonics in Infection Research (LPI), Albert-Einstein-Straße 9, 07745 Jena, Germany

*Corresponding author: eugen@blink-dx.com

**Table of Contents**

Page S3: Table S1 Fluorescent dye labels and the respective relative dye levels of the 16plex femNRB set. femNRB codes sorted by Cy3- and Cy7-core (chitosan) and shell (agarose) levels

Page S4: Table S2 femNRBs and their associated PCR assays

Page S5: Table S3 List of genetic material used in this work

Page S6: Table S4 Samples containing respective nominal targets (copies/sample) used for generating data set 0

Page S7: Table S5 Samples containing respective nominal targets (copies/sample) used for generating data set 1

Page S8: Table S6 Samples containing respective nominal targets (copies/sample) used for generating data set 2

Page S9: Table S7 Samples containing respective nominal targets (copies/sample) used for generating data set 3

Page S10: Table S8 Samples containing respective nominal targets (copies/sample) used for generating data set 4

Page S11: Table S9 Samples containing respective nominal targets (copies/sample) used for the gapA target, a similar setup was used for ddl and sesC in data set 5

Page S12: Table S10 Samples containing respective targets (copies/sample) used for generating data set 6 based on ddPCR reference method. Sample 4 is a mixture of sample 1, 2 and 3

Page S13: Table S11 Bhattacharyya coefficients, Bead validity and Bead count across all experiments

Page S14: Figure S1 Flowchart of Data Analysis Workflow

Page S15: Figure S2 Flow chart of bead decoding procedures

Page S16: Figure S3 Location of the bead decoding features of all valid beads for an example experiment with (A) 10 and (B) 16 bead types.

Page S17: Figure S4 dPCR measuring range for different multiplex levels utilizing 25,000 beads

Page S18: Figure S5 Binding kinetics for *S. aureus* DNA in three different fragment lengths and at two different concentrations

Page S19: Table S12 DNA binding time, estimated t-values at 90 and 99 with 95% confidence intervals and coefficient of determination ($R^2$)

Page S20: Figure S6 results of agarose gel electrophoresis for individual DNAs after ultra-sonic treatment as used for the study

Page S21: Table S13 Precision results of the nine targets at low DNA samples (20,000 input copies) based on data set

Page S22: Table S14 Precision results of the nine targets based on all available results for high DNA samples (150,000 input copies) for data sets 2, 4 and 5

Page S23: Table S15 Comparative overview of multiplexing methodologies in digital PCR

Table S1 Fluorescent dye labels and the respective relative dye levels of the 16-plex femNRB set. femNRB codes sorted by Cy3- and Cy7-core (chitosan) and shell (agarose) levels

| femNRB Code | Coding Scheme | Cy3-labeled agarose, factor | Cy7-labeled agarose, factor | Cy3-labeled chitosan, factor | Cy7-labeled chitosan, factor |
|---|---|---|---|---|---|
| 3 | S0001-C0000 | 0 | 1 | 0 | 1.0 |
| 14 | S0001-C0001 | 0 | 1 | 0 | 25.4 |
| 5 | S0001-C0100 | 0 | 1 | 2.8 | 1.0 |
| 16 | S0001-C0101 | 0 | 1 | 3.0 | 25.4 |
| 1 | S0100-C0000 | 1 | 0 | 1.0 | 0.0 |
| 6 | S0100-C0001 | 1 | 0 | 1.0 | 1.5 |
| 4 | S0101-C0000 | 1 | 1 | 1.0 | 1.0 |
| 10 | S0101-C0001 | 1 | 1 | 1.0 | 25.4 |
| 2 | S0100-C0100 | 1 | 0 | 18.0 | 0.0 |
| 7 | S0100-C0101 | 1 | 0 | 18.0 | 1.5 |
| 12 | S0101-C0100 | 1 | 1 | 18.0 | 1.0 |
| 15 | S0101-C0101 | 1 | 1 | 20.0 | 25.4 |
| 8 | S0200-C0000 | 6.4 | 0 | 5.8 | 0.0 |
| 9 | S0200-C0001 | 6.4 | 0 | 5.2 | 1.5 |
| 11 | S0201-C0000 | 6.4 | 1 | 5.2 | 1.0 |
| 13 | S0201-C0001 | 6.4 | 1 | 5.2 | 25.4 |

Table S1 femNRBs and their associated PCR assays

| Bead Code | Targe/ Gene | Primers/ Probe | Description | Coding Scheme |
|---|---|---|---|---|
| 1 | aac6 | fw: TCGGCAGAAGAAGTAGAAGA<br>rv: ACTAATGGATGCAATTCCCAA<br>p: ATTGGTGCAATCCCTCAATACGGTATCACA | *E. faecium* species marker | S0100-C0000 |
| 2 | basC | fw: CATCAGTTTTATTATTACGACAGTTTT<br>rv: GGTAATTGTTTTGAAGCCCA<br>p: CCACGCCGTGAATATGACCATTATTG | *A. baumannii* species marker | S0100-C0100 |
| 3 | BG | fw: TCCACCGAACAATCCGATC<br>rv: GCGGCAAACACGGAGAAA<br>p: CCGATTCACAGACAAGCTCCGTCATTTGATC | *B. atrophaeus* species marker | S0001-C0000 |
| 4 | Cfa | fw: CTGGGACATTCAACTTCATC<br>rv: TCAGGATTTTGCAGAACAGAA<br>p: TAGGGCTTGGCGAAAGCTATATGGAA | *C. freundii* species marker | S0101-C0000 |
| 5 | Ddl | fw: TTAGGAAATGAAGATGTCCGTAC<br>rv: GCTACTTCTTCTGGAACATGC<br>p: TTACCTGGTGAAGTGGTGAAAGATGTCG | *E. faecalis* species marker | S0001-C0100 |
| 6 | ecfX | fw: ATGAGCGCTTCCGTGGTTC<br>rv: AGGAAGCGCAGCAACTCG<br>p: TCTCGCATGCCTATCAGGCGTTCCAT | *P. aeruginosa* species marker | S0100-C0001 |
| 7 | gapA | fw: GGTGACTTAAAAACAATCGTATTCA<br>rv: CTTCAACTAAACCAAAGTCATCG<br>p: GGTTCTGAAACAGTTGTTTCAGGTGCTTCA | *S. aureus* species marker | S0100-C0101 |
| 8 | Khe | fw: GGTTTACGTCTCAACCGG<br>rv: AGAGATAGCCGTTTATCCACAC<br>p: TGAGGAAGAGTTCATCTACGTGCTGGAGGG | *K. pneumoniae* species marker | S0200-C0000 |
| 9 | sesC | fw: GTGTCTACCTCAAGCTGTCATG<br>rv: TTGGATTTTGTCAGCGATG<br>p: TTAGTGGTTCGCTGGTTGGTTATGGCTT | *S. epidermidis* species marker | S0200-C0001 |
| 10 | rpp30 | fw: GCCAAATTCTGCTCGTTGTTAG<br>rv: CTTCCCTCACGGCATATACTTC<br>p: TCACCAGCTGGATGTCCACATTCA | Human single copy gene as binding and PCR control | S0101-C0001 |
| 11 | NDM | fw: GGTTTGATCGTCAGGGATG<br>rv: GACCGGCAGGTTGATCT<br>p: ATGACCAGACCGCCCAGATCCTCA | New Delhi Metallo-Beta-Lactamase (Carbapenemase) | S0201-C0000 |
| 12 | OXA181 | fw: GCTACCCAGCAAATCGC<br>rv: ATAGTCGCCATTGGCTTCG<br>p: CAACAAGCTGCACGTTTCTGAGCGTAG | OXA48-like Serin-Protease (Carbapenemase) | S0101-C0100 |
| 13 | blaCTX-M1/15 | fw: CAGTTCACGCTGATGGC<br>rv: CGACTGCCGCTCTAATTC<br>p: ACCGTCACGCTGTTGTTAGGAAGTGT | Extended spectrum β-lactamase-producing Enterobacteriaceae (ESBL) | S0201-C0001 |
| 14 | blaZ | fw: GTGTTCCAAAAGACTATAAGGTTGC<br>rv: TTCAGATTGGCCCTTAGG<br>p: GTGGTCAAGCAATAACATATGCTTCTAGA | Beta-Lactamase (Penicillin resistance) | S0001-C0001 |
| 15 | mecA | fw: TGGCATGAGTAACGAAGAATATAA<br>rv: GAGTTGAACCTGGTGAAGTTG<br>p: AAAGAACCTCTGCTCAACAAGTTCCAGA | Penicillin-binding protein PBP2a (Methicillin resistance) | S0101-C0101 |
| 16 | blaVIM | fw: GGCAACGTACGCATCAC<br>rv: GCAGCACCGGGATAGAA<br>p: TCTCTAGAAGGACTCTCATCGAGCGGG | Verona-Integron-encoded Metallo-β-Lactamase (Carbapenemase) | S0001-C0101 |

Table S2 List of genetic material used in this work

| Targets | Species | Strain |
|---|---|---|
| *aac6* | *Enterococcus faecium* | IPHT: UK040 |
| *basC, blaVIM* | *Acinetobacter baumannii* | IPHT: CARB128 |
| *BG* | *Bacillus atrophaeus* | DSM 2277 |
| *Cfa* | *Citrobacter freundii* | IPHT: BL77 |
| *Ddl* | *Enterococcus faecalis* | IPHT: UK045 |
| *ecfX* | *Pseudomonas aeruginosa* | IPHT: UKJ55_UR23181 |
| *gapA, mecA, blaZ* | *Staphylococcus aureus* | MW2_R27 CP194231.1 |
| *khe, blaNDM, blaOXA-48-like, blaCTX-M1/15* | *Klebsiella pneumoniae* | IPHT: Nord77 |
| *sesC* | *Staphylococcus epidermidis* | ATCC35984 |
| *rpp30* | Homo sapiens | from WBC isolation |

Table S3 Samples containing respective nominal targets (copies/sample) used for generating data set 0

| | **Samples** | | | | | | | | |
|---|---|---|---|---|---|---|---|---|---|
| **Targets** | **1** | **2** | **3** | **4** | **5** | **6** | **7** | **8** | **9** |
| AAC6 | 20,000 | 0 | 0 | 0 | 0 | 0 | 0 | 0 | 0 |
| basC | 0 | 20,000 | 0 | 0 | 0 | 0 | 0 | 0 | 0 |
| BG | 0 | 0 | 20,000 | 0 | 0 | 0 | 0 | 0 | 0 |
| cfa | 0 | 0 | 0 | 20,000 | 0 | 0 | 0 | 0 | 0 |
| ddl | 0 | 0 | 0 | 0 | 20,000 | 0 | 0 | 0 | 0 |
| ecfX | 0 | 0 | 0 | 0 | 0 | 20,000 | 0 | 0 | 0 |
| gapA | 0 | 0 | 0 | 0 | 0 | 0 | 20,000 | 0 | 0 |
| Khe | 0 | 0 | 0 | 0 | 0 | 0 | 0 | 20,000 | 0 |
| sesC | 0 | 0 | 0 | 0 | 0 | 0 | 0 | 0 | 20,000 |
| rpp30 | 100,000 | 100,000 | 100,000 | 100,000 | 100,000 | 100,000 | 100,000 | 100,000 | 100,000 |

Table S5 Samples containing respective nominal targets (copies/sample) used for generating data set 1

| | Samples | | | | | | | | |
|---|---|---|---|---|---|---|---|---|---|
| **Targets** | **1** | **2** | **3** | **4** | **5** | **6** | **7** | **8** | **9** |
| AAC6 | 150,000 | 0 | 0 | 0 | 0 | 0 | 0 | 0 | 0 |
| basC | 0 | 150,000 | 0 | 0 | 0 | 0 | 0 | 0 | 0 |
| BG | 0 | 0 | 150,000 | 0 | 0 | 0 | 0 | 0 | 0 |
| cfa | 0 | 0 | 0 | 150,000 | 0 | 0 | 0 | 0 | 0 |
| ddl | 0 | 0 | 0 | 0 | 150,000 | 0 | 0 | 0 | 0 |
| ecfX | 0 | 0 | 0 | 0 | 0 | 150,000 | 0 | 0 | 0 |
| gapA | 0 | 0 | 0 | 0 | 0 | 0 | 150,000 | 0 | 0 |
| Khe | 0 | 0 | 0 | 0 | 0 | 0 | 0 | 150,000 | 0 |
| sesC | 0 | 0 | 0 | 0 | 0 | 0 | 0 | 0 | 150,000 |
| rpp30 | 100,000 | 100,000 | 100,000 | 100,000 | 100,000 | 100,000 | 100,000 | 100,000 | 100,000 |

Table S6 Samples containing respective nominal targets (copies/sample) used for generating data set 2

| | **Samples** | | | | | | | | |
|---|---|---|---|---|---|---|---|---|---|
| **Targets** | **1** | **2** | **3** | **4** | **5** | **6** | **7** | **8** | **9** |
| AAC6 | 20,000 | 150,000 | 150,000 | 150,000 | 150,000 | 150,000 | 150,000 | 150,000 | 150,000 |
| basC | 150,000 | 20,000 | 150,000 | 150,000 | 150,000 | 150,000 | 150,000 | 150,000 | 150,000 |
| BG | 150,000 | 150,000 | 20,000 | 150,000 | 150,000 | 150,000 | 150,000 | 150,000 | 150,000 |
| cfa | 150,000 | 150,000 | 150,000 | 20,000 | 150,000 | 150,000 | 150,000 | 150,000 | 150,000 |
| ddl | 150,000 | 150,000 | 150,000 | 150,000 | 20,000 | 150,000 | 150,000 | 150,000 | 150,000 |
| ecfX | 150,000 | 150,000 | 150,000 | 150,000 | 150,000 | 20,000 | 150,000 | 150,000 | 150,000 |
| gapA | 150,000 | 150,000 | 150,000 | 150,000 | 150,000 | 150,000 | 20,000 | 150,000 | 150,000 |
| Khe | 150,000 | 150,000 | 150,000 | 150,000 | 150,000 | 150,000 | 150,000 | 20,000 | 150,000 |
| sesC | 150,000 | 150,000 | 150,000 | 150,000 | 150,000 | 150,000 | 150,000 | 150,000 | 20,000 |
| rpp30 | 100,000 | 100,000 | 100,000 | 100,000 | 100,000 | 100,000 | 100,000 | 100,000 | 100,000 |

Table S7 Samples containing respective nominal targets (copies/sample) used for generating data set 3

| | **Samples** | | | |
|---|---|---|---|---|
| **Targets** | **1** | **2** | **3** | **4** |
| AAC6 | 20,000 | 20,000 | 20,000 | 20,000 |
| basC | 20,000 | 20,000 | 20,000 | 20,000 |
| BG | 20,000 | 20,000 | 20,000 | 20,000 |
| cfa | 20,000 | 20,000 | 20,000 | 20,000 |
| ddl | 20,000 | 20,000 | 20,000 | 20,000 |
| ecfX | 20,000 | 20,000 | 20,000 | 20,000 |
| gapA | 20,000 | 20,000 | 20,000 | 20,000 |
| Khe | 20,000 | 20,000 | 20,000 | 20,000 |
| sesC | 20,000 | 20,000 | 20,000 | 20,000 |
| rpp30 | 100,000 | 100,000 | 100,000 | 100,000 |

Table S8 Samples containing respective nominal targets (copies/sample) used for generating data set 4

| | Samples | | | | | | | | |
|---|---|---|---|---|---|---|---|---|---|
| **Target** | **1** | **2** | **3** | **4** | **5** | **6** | **7** | **8** | **9** |
| AAC6 | 0 | 150,000 | 150,000 | 150,000 | 150,000 | 150,000 | 150,000 | 150,000 | 150,000 |
| basC | 150,000 | 0 | 150,000 | 150,000 | 150,000 | 150,000 | 150,000 | 150,000 | 150,000 |
| BG | 150,000 | 150,000 | 0 | 150,000 | 150,000 | 150,000 | 150,000 | 150,000 | 150,000 |
| cfa | 150,000 | 150,000 | 150,000 | 0 | 150,000 | 150,000 | 150,000 | 150,000 | 150,000 |
| ddl | 150,000 | 150,000 | 150,000 | 150,000 | 0 | 150,000 | 150,000 | 150,000 | 150,000 |
| ecfX | 150,000 | 150,000 | 150,000 | 150,000 | 150,000 | 0 | 150,000 | 150,000 | 150,000 |
| gapA | 150,000 | 150,000 | 150,000 | 150,000 | 150,000 | 150,000 | 0 | 150,000 | 150,000 |
| Khe | 150,000 | 150,000 | 150,000 | 150,000 | 150,000 | 150,000 | 150,000 | 0 | 150,000 |
| sesC | 150,000 | 150,000 | 150,000 | 150,000 | 150,000 | 150,000 | 150,000 | 150,000 | 0 |
| rpp30 | 100,000 | 100,000 | 100,000 | 100,000 | 100,000 | 100,000 | 100,000 | 100,000 | 100,000 |

Table S9 Samples containing respective nominal targets (copies/sample) used for the gapA target, a similar setup was used for ddl and sesC in data set 5

| | **Samples** | | | | | |
|---|---|---|---|---|---|---|
| **Target** | **1** | **2** | **3** | **4** | **5** | **6** |
| AAC6 | 150,000 | 150,000 | 150,000 | 150,000 | 150,000 | 150,000 |
| basC | 150,000 | 150,000 | 150,000 | 150,000 | 150,000 | 150,000 |
| BG | 150,000 | 150,000 | 150,000 | 150,000 | 150,000 | 150,000 |
| Cfa | 150,000 | 150,000 | 150,000 | 150,000 | 150,000 | 150,000 |
| ddl | 150,000 | 150,000 | 150,000 | 150,000 | 150,000 | 150,000 |
| ecfX | 150,000 | 150,000 | 150,000 | 150,000 | 150,000 | 150,000 |
| gapA | 2,000 | 10,000 | 20,000 | 50,000 | 160,000 | 250,000 |
| Khe | 150,000 | 150,000 | 150,000 | 150,000 | 150,000 | 150,000 |
| sesC | 150,000 | 150,000 | 150,000 | 150,000 | 150,000 | 150,000 |
| rpp30 | 100,000 | 100,000 | 100,000 | 100,000 | 100,000 | 100,000 |

Table S10 Samples containing respective targets (copies/sample) used for generating data set 6 based on ddPCR reference method. Sample 4 is a mixture of sample 1, 2 and 3

| | **Samples** | | | |
|---|---|---|---|---|
| **Target** | **1** | **2** | **3** | **4** |
| AAC6 | 0 | 0 | 0 | 0 |
| BasC | 19,834 | 0 | 0 | 19,834 |
| BG | 0 | 0 | 0 | 0 |
| Cfa | 0 | 0 | 0 | 0 |
| Ddl | 0 | 0 | 0 | 0 |
| ecfX | 0 | 0 | 0 | 0 |
| gapA | 0 | 15,345 | 0 | 15,345 |
| Khe | 0 | 0 | 6,185 | 6,184 |
| sesC | 0 | 0 | 0 | 0 |
| rpp30 | 105,501 | 105,501 | 105,501 | 105,501 |
| NDM | 0 | 0 | 0 | 0 |
| OXA181 | 0 | 0 | 214,840 | 214,840 |
| CTX-M1 | 0 | 0 | 24,696 | 24,696 |
| blaZ | 0 | 76,079 | 0 | 76,079 |
| mecA | 0 | 16,832 | 0 | 16,832 |
| VIM | 2,455 | 0 | 0 | 2,455 |

Table S11 Bhattacharyya coefficients, bead validity and bead count across all experiments

| Plex | Bhattacharyya Coefficient [-] | | | | | Bead Validity [%] | | | Bead Count per Code [-] |
|---|---|---|---|---|---|---|---|---|---|
| | median | 1/median | IQR | max | 1/max | mean | min | max | mean |
| **10** | 1.07E-08 | 93,870,000 | 4.47E-08 | 0.000002 | 430,000 | 89.53 | 79.07 | 93.67 | 1,899 |
| **16** | 7.85E-08 | 12,750,000 | 3.78E-07 | 0.000007 | 140,000 | 89.22 | 84.72 | 91.45 | 1,229 |
| **all** | 1.47E-08 | 67,970,000 | 5.94E-08 | 0.000007 | 140,000 | 89.50 | 79.07 | 93.67 | - |

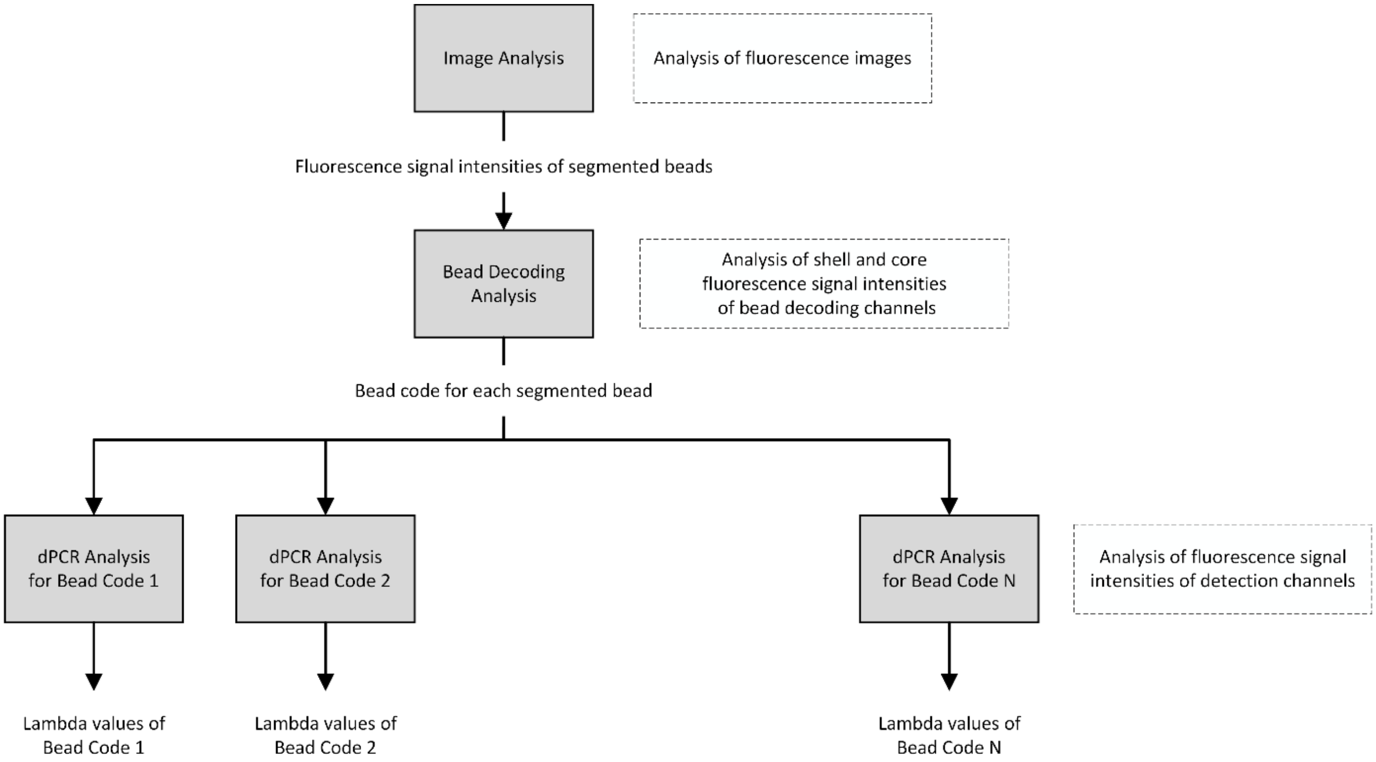


Figure S1 Flowchart of data analysis workflow

In the first step, the fluorescence signal intensities for the bead shell and core components of bead decoding and detection channels are determined. In the subsequent bead decoding step, the bead code is assigned to each individual bead based on the shell and core intensities of the bead decoding channels. This analysis is performed for each well individually. In the third step, the Poisson analysis is conducted separately for the valid beads of each bead code in each well by analyzing respective fluorescence signal intensities of detection channels.

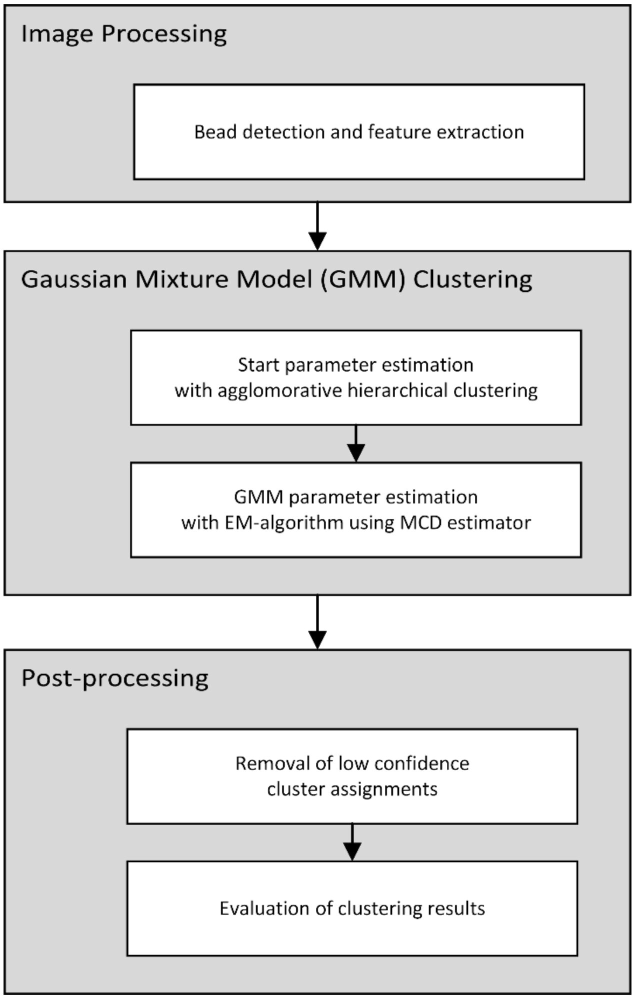


Figure S2 Flow chart of bead decoding procedures

Beads are first detected in the input images, and relevant features are extracted. A Gaussian Mixture Model (GMM) is then applied for clustering, with two modifications to the standard approach: (1) initial parameters are estimated using agglomerative clustering on a log-transformed subset of the data, and (2) the EM-algorithm employs the MCD covariance estimator to ensure robustness against outliers. During post-processing, low-confidence inter- and intra-cluster assignments are removed, and clustering performance is evaluated using the Bhattacharyya coefficients.

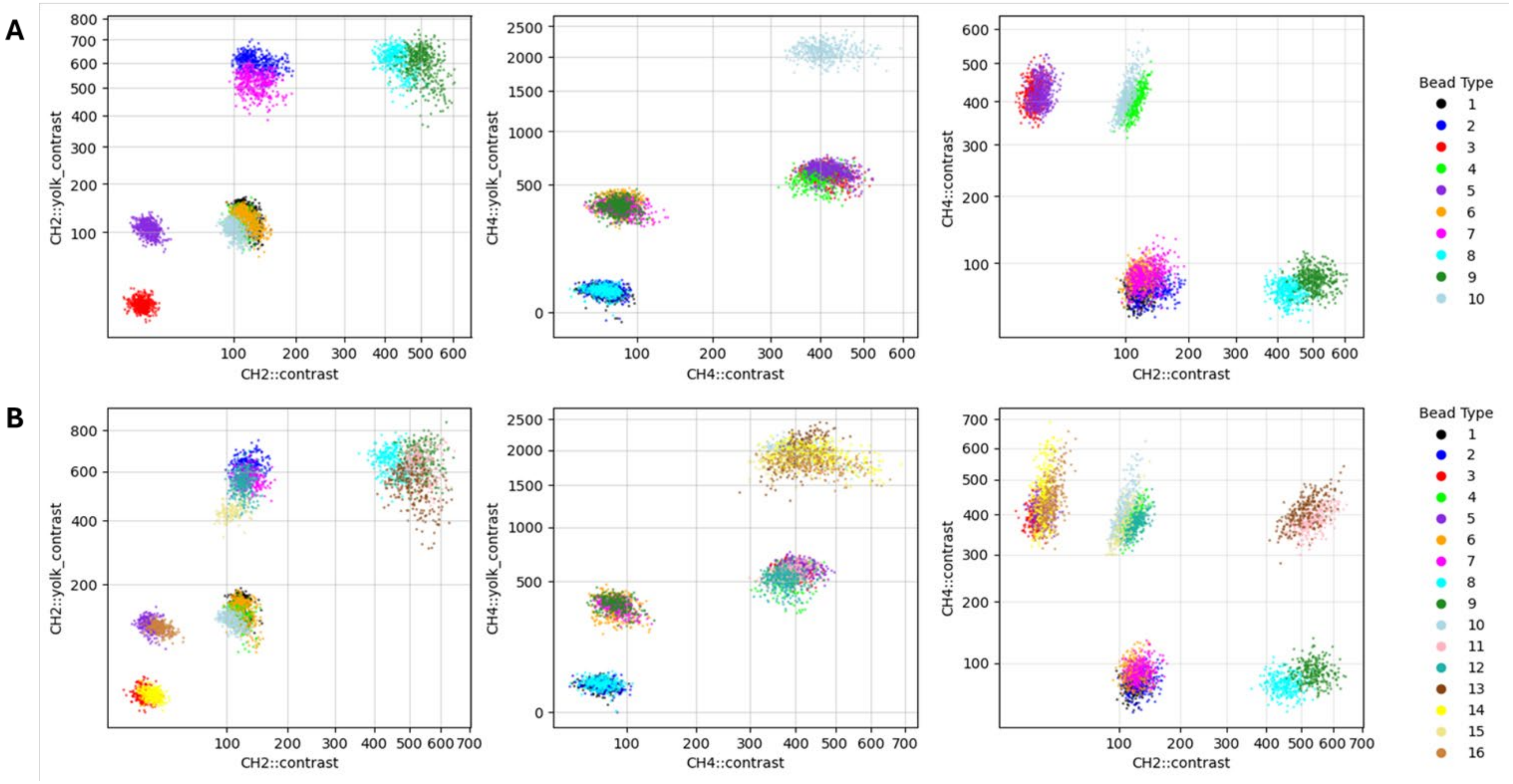


Figure S3 Location of the bead decoding features of all valid beads for an example experiment with (A) 10 and (B) 16 bead types.

The corresponding Bhattacharyya coefficients were $5.25\text{x}10^{-8}$ for the 10-plex assay and $7.12\text{x}10^{-6}$ for the 16-plex assay, equivalent to approximately one misclassified bead in 19,050,000 and 140,000, respectively.

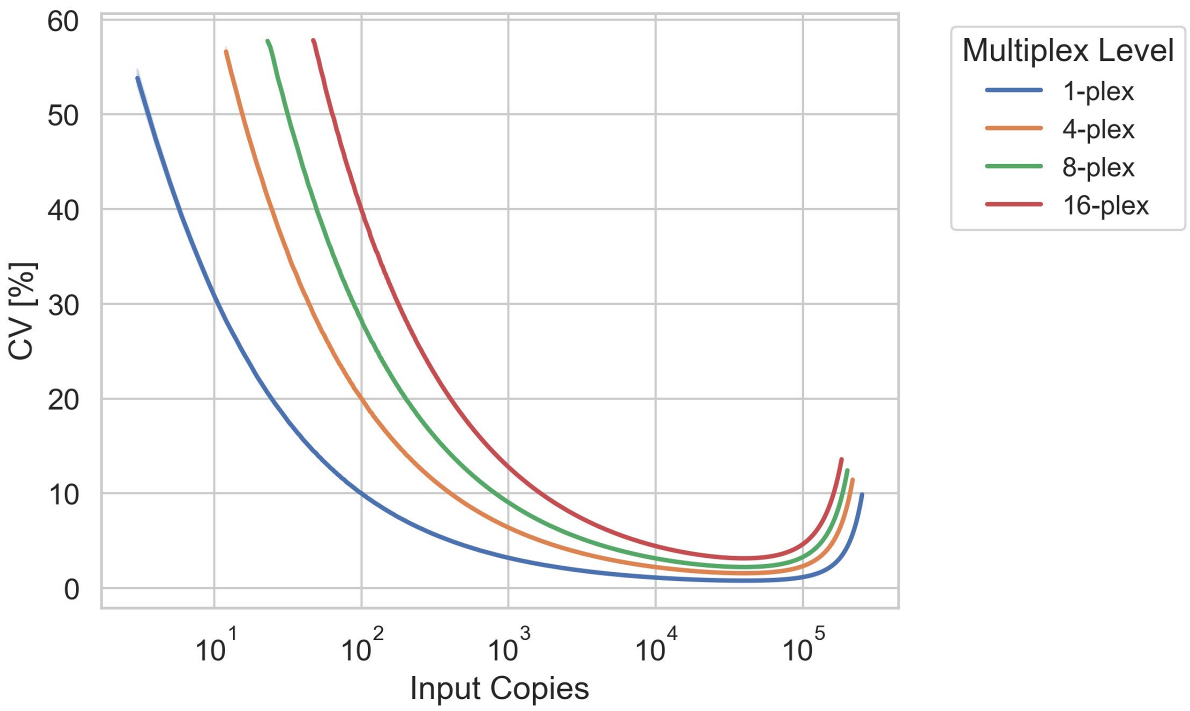


Figure S4 dPCR measuring range for different multiplex levels utilizing 25,000 beads. Start and end points of the lines mark the lower and upper limits of the maximum achievable dynamic range.

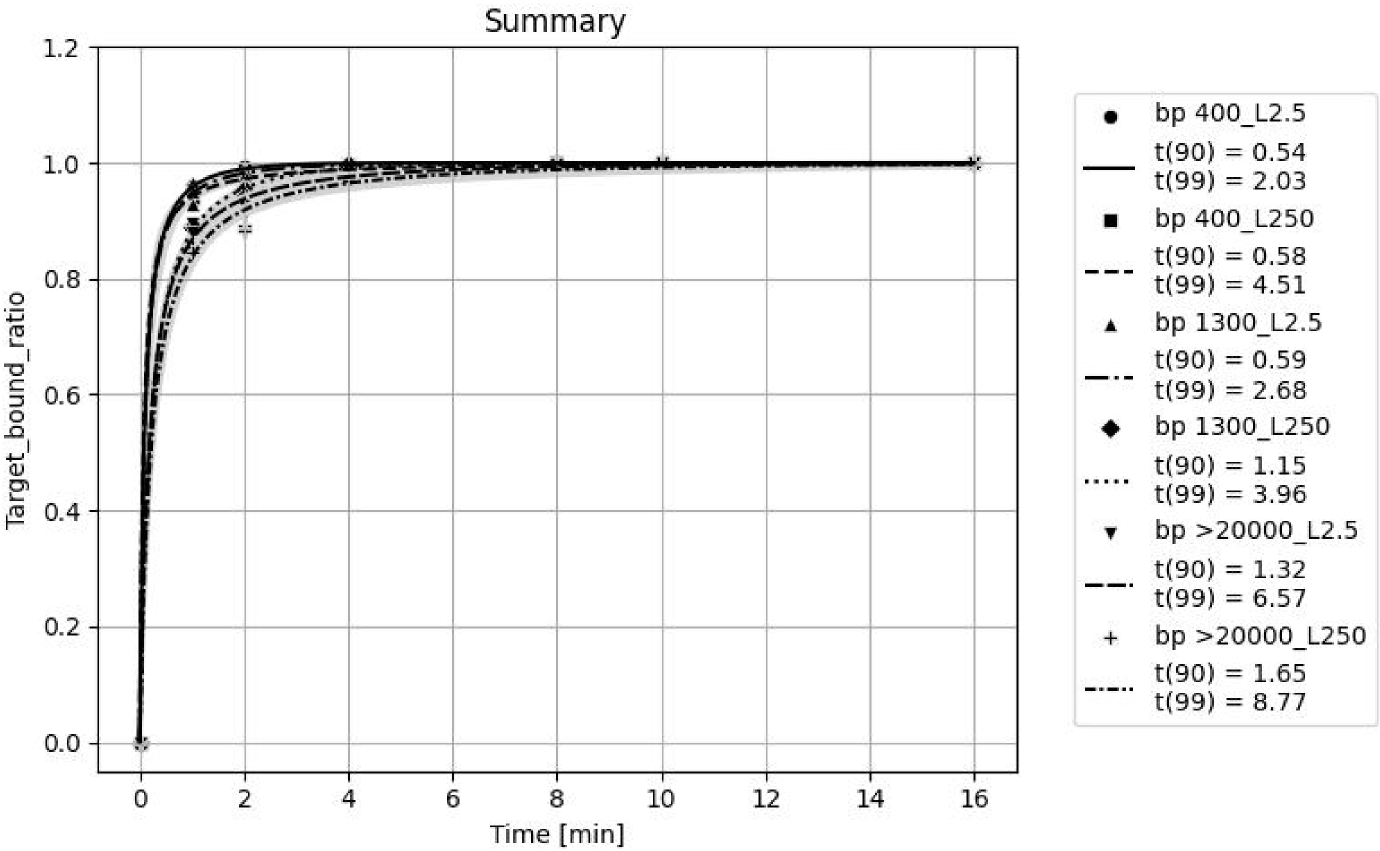


Figure S5 Binding kinetics for *S. aureus* DNA in three different fragment lengths (~400bp, ~1300bp as used in the multiplex experiments, >20000bp) at two different concentrations N1= 4.48x$10^4$ copies, λ=2.5 and N2=4.48x$10^6$ copies, λ=250 applied to 20,000 femNRBs in 80µl binding mix (250 beads/µl). The selected concentrations were chosen to cover the range of DNA inputs used in the present study. Figure S5 shows the experimental data together with the corresponding generalized second order adsorption model-based fits, depicted as solid black and dashed black lines. Data are presented as a fraction of total input DNA captured by the beads. The times required to reach 90% and 99% binding (t90 and t99, respectively) are indicated for each sample.

Table S12 DNA binding time, estimated t-values at 90% and 99% binding with 95% confidence intervals and coefficient of determination ($R^2$).

| **Sample** | **t(90) with 95%CI [min]** | **t(99) with 95%CI [min]** | **$R^2$** |
|---|---|---|---|
| bp 400_L2.5 | 0.544 [0.488, 0.600] | 2.032 [1.821, 2.243] | 1.000 |
| bp 400_L250 | 0.580 [0.498, 0.663] | 4.511 [3.160, 5.862] | 0.999 |
| bp 1300_L2.5 | 0.587 [0.456, 0.718] | 2.685 [1.802, 3.567] | 0.999 |
| bp 1300_L250 | 1.153 [1.093, 1.213] | 3.964 [3.203, 4.725] | 0.999 |
| bp 20000_L2.5 | 1.323 [1.178, 1.467] | 6.569 [3.564, 9.574] | 0.996 |
| bp 20000_L250 | 1.652 [1.472, 1.832] | 8.766 [4.365, 13.168] | 0.995 |

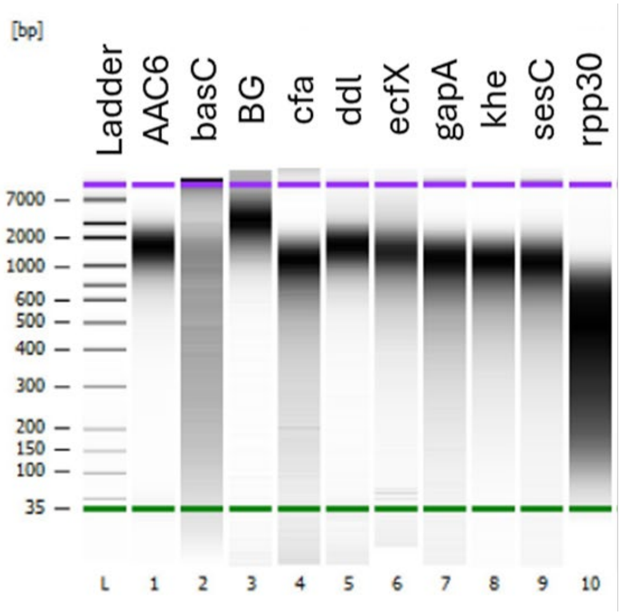


| DNA | bp range | Peak bp |
|---|---|---|
| AAC6 | 400-5000 | 1800 |
| basC | 150-2000 | 1600 |
| BG | 1000-10000 | 3500 |
| cfa | 500-3000 | 1300 |
| ddl | 600-5000 | 1600 |
| ecfX | 500-5500 | 1500 |
| gapA | 550-3000 | 1300 |
| khe | 500-3000 | 1200 |
| sesC | 500-3500 | 1300 |
| rpp30 | 100-800 | 500 |

Figure S6 results of agarose gel electrophoresis for individual DNAs after ultra-sonic treatment as used for the study

Table S13 Precision results of the nine targets at low DNA samples (20,000 input copies) based on data set 3

| Analyte | No. Exp | No. data sets | Lambda [cp/Bead] mean | Copies in Sample [cp] mean | Lambda [cp/Bead] std | Lambda [cp/Bead] cv% |
|---|---|---|---|---|---|---|
| AAC6 | 11 | 1 | 0.212 | 21,163 | 0.0162 | 7.63 |
| BG | 11 | 1 | 0.191 | 19,065 | 0.0176 | 9.22 |
| basC | 11 | 1 | 0.226 | 22,553 | 0.0153 | 6.77 |
| cfa | 11 | 1 | 0.207 | 20,709 | 0.0168 | 8.12 |
| ddl | 11 | 1 | 0.191 | 19,149 | 0.0177 | 9.24 |
| ecfX | 11 | 1 | 0.201 | 20,071 | 0.0104 | 5.20 |
| gapA | 11 | 1 | 0.186 | 18,593 | 0.018 | 9.69 |
| khe | 11 | 1 | 0.208 | 20,841 | 0.0188 | 9.03 |
| sesC | 11 | 1 | 0.207 | 20,713 | 0.0099 | 4.78 |

Table S14 Precision results of the nine targets based on all available results for high DNA samples (150,000 input copies) for data sets 2, 4 and 5

| Analyte | No. Exp | No. data sets | Lambda [cp/Bead] mean | Copies in Sample [cp] mean | Lambda [cp/Bead] std | Lambda [cp/Bead] CV% |
|---|---|---|---|---|---|---|
| AAC6 | 59 | 3 | 1.647 | 164,727 | 0.0619 | 3.76 |
| BG | 59 | 3 | 1.445 | 144,490 | 0.0782 | 5.41 |
| basC | 59 | 3 | 1.729 | 172,922 | 0.0568 | 3.29 |
| cfa | 59 | 3 | 1.538 | 153,800 | 0.0512 | 3.33 |
| ddl | 59 | 3 | 1.506 | 150,572 | 0.0685 | 4.55 |
| ecfX | 60 | 3 | 1.562 | 156,158 | 0.0488 | 3.13 |
| gapA | 47 | 2 | 1.516 | 151,557 | 0.073 | 4.82 |
| khe | 59 | 3 | 1.557 | 155,651 | 0.0571 | 3.67 |
| esC | 59 | 3 | 1.551 | 155,102 | 0.0676 | 4.36 |

Table S15 Comparative overview of multiplexing methodologies in digital PCR

| Multiplexing Approach | Mechanism & Characteristics | Advantages | Limitations | Exemplary Reference |
|---|---|---|---|---|
| Fluorophore-based | Each target is assigned a distinct fluorescent dye, and detection relies on spectral separation. The method is robust, well-established, and compatible with standard dPCR/qPCR instruments. | Simple, mature, widely supported; high robustness; straightforward interpretation. | Strongly limited by available channels; spectral bleed-through; expensive labeled probes. Primer interference. | de Korne-Elenbaas J. Design, validation, and implementation of multiplex digital PCR assays. Lett Appl Microbiol. 2025. DOI: 10.1093/lambio/ovae137 |
| Amplitude-based | Probe concentrations are tuned to generate distinct fluorescence amplitude clusters within a single channel. This enables multiplexing without additional dyes but requires careful optimization to avoid cluster overlap. | Cost-effective; no need for extra dyes; expands multiplexing on single-channel systems. | Cluster overlap risk; sensitive to assay balance; reduced robustness in complex samples. Primer interference. | Hussung S. Multiplex detection of KRAS mutations using amplitude multiplexing in ddPCR. Clin Chem Lab Med. 2020. DOI: 10.1515/cclm-2020-0233 |
| Algorithmic (ML-based decoding) | Machine-learning algorithms classify droplets or partitions using multidimensional fluorescence features. This enables plex levels beyond human-interpretable clusters. | Very high plex potential; adaptable to complex signal patterns; scalable. | Requires ML expertise and extensive training data, QC pipelines, and computational resources. Risk of overfitting; computational overhead; QC complexity. | Alfonso De Falco, Digital PCR cluster predictor: a universal R-package and shiny app for the automated analysis of multiplex digital PCR data, Bioinformatics, 2023, DOI: 10.1093/bioinformatics/btad282 |
| Immobilized primers | Primers are covalently immobilized at defined spatial positions (arrays, gel pads, bead surfaces), creating a physical assay code. Amplification occurs only where the corresponding primers are fixed. | Ultra-high plex; no spectral limitations; stable spatial encoding. | Complex manufacturing; low flexibility; inefficient amplification due to immobilized reagents, challenging to redesign assays | Strizhkov BN. PCR amplification on microarrays of gel-immobilized oligonucleotides. BioTechniques. 2000. DOI: 10.2144/00292bm03 |

| | | | | |
|---|---|---|---|---|
| Melt-curve multiplexing | Targets are distinguished by their characteristic melting temperatures using a single fluorescent dye. Digital melt-curve analysis enables high plexing without additional fluorophores. | High plex without extra dyes; cost-effective; compatible with simple optics. | Requires precise temperature control; overlapping Tm clusters reduce plex; slower workflows. Primer interference. | Yu H. Multiplex digital PCR with melting curve analysis on a SlipChip. Analyst. 2022. DOI: 10.1039/D1AN01916C |
| Bead/ Nanowell-Combo | Each bead population carries a unique primer set and fluorescent code, enabling target-specific reactions within individual beads. Beads act both as binders and as primer carriers. After individual partitioning into nanowells, the beads undergo PCR, and the bead-specific code reveals which target has been amplified. | Highly scalable; flexible assay design; no primer interference. | Targe binding efficiency can be different for different primer sets. Requires sophisticated micro structured nanowell-substates for bead partitioning, number of different | Henley, W. H. Spatially Isolated Reactions in a Complex Array: Using Magnetic Beads to Purify and Quantify Nucleic Acids with Digital and Quantitative Real-Time PCR in Thousands of Parallel Microwells. Lab on a Chip 2020 DOI: 10.1039/d0lc00069h |
| Spatially isolated rehydration | Assay-specific primers or reagents are dried into separate microchambers and rehydrated only after sample partitioning. This prevents primer interactions and ensures clean multiplexing. | No primer interference; robust multiplexing; excellent assay separation | Limited by chip fabrication; low flexibility; requires precise drying/rehydration control; number of compartments for each primer/probe sets; achievable limit of detection increases with the number of different primer/probe sets | Xie Y. Controlled rehydration of dried reagents for robust multiplex digital PCR. Anal Chem. 2022. DOI: 10.1021/acs.analchem.2c00345 |
| Nanoreactor Beads | Differentially fluorescence encoded magnetic nanoreactor beads with pre-bound primer/probes are used for partitioning by binding and subsequent target specific PCR amplification and detection. | No primer interference; robust multiplexing; excellent assay separation, highly flexible, simple disposable architecture | Limited by the number of available fluorescence codes, the achievable limit of detection increases with the number of codes. | this work |
